# A Comprehensive Understanding of the Genomic Bone Tumor Landscape: A Multicenter Prospective Study

**DOI:** 10.3389/fonc.2022.835004

**Published:** 2022-06-08

**Authors:** Xianbiao Xie, Yiying Bian, Haomiao Li, Junqiang Yin, Lantian Tian, Renbing Jiang, Ziliang Zeng, Xiaoliang Shi, Zixiong Lei, Changhe Hou, Yueting Qu, Liwei Wang, Jingnan Shen

**Affiliations:** ^1^Department of Musculoskeletal Oncology Center, The First Affiliated Hospital of Sun Yat-sen University, Guangzhou, China; ^2^Department of Musculoskeletal Oncology, Center for Orthopaedic Surgery, The Third Affiliated Hospital of Southern Medical University, Guangzhou, China; ^3^Department of Hepatobiliary Surgery, Affiliated Hospital of Qingdao University, Qingdao, China; ^4^Department of Bone and Soft Tissue, Affiliated Tumor Hospital of Xinjiang Medical University, Urumqi, China; ^5^Department of Medicine, Shanghai OrigiMed Co., Ltd, Shanghai, China

**Keywords:** bone tumor, next-generation sequencing, diagnosis, gene fusion, biomarker

## Abstract

Complexity and heterogeneity increases the difficulty of diagnosis and treatment of bone tumors. We aimed to identify the mutational characterization and potential biomarkers of bone tumors. In this study, a total of 357 bone tumor patients were recruited and the next generation sequencing (NGS)-based YuanSu450 panel, that includes both DNA and RNA sequencing, was performed for genomic alteration identification. The most common mutated genes in bone tumors included *TP53*, *NCOR1*, *VEGFA*, *RB1*, *CCND3*, *CDKN2A*, *GID4, CCNE1*, *TERT*, and *MAP2K4*. The amplification of genes such as *NCOR1*, *VEGFA*, and *CCND3* mainly occurred in osteosarcoma. Germline mutation analysis reveal a high frequency of HRD related mutations (46.4%, 13/28) in this cohort. With the assistance of RNA sequencing, 16.8% (19/113) gene fusions were independently detected in 20% (16/79) of patients. Nearly 34.2% of patients harbored actionable targeted mutations, of which the most common mutation is *CDKN2A* deletion. The different mutational characterizations between juvenile patients and adult patients indicated the potential effect of age in bone tumor treatment. According to the genomic alterations, the diagnosis of 26 (7.28%) bone tumors were corrected. The most easily misdiagnosed bone tumor included malignant giant cell tumors of bone (2.8%, 10/357) and fibrous dysplasia of bone (1.7%, 6/357). Meanwhile, we found that the mutations of *MUC16* may be a potential biomarker for the diagnosis of mesenchymal chondrosarcomas. Our results indicated that RNA sequencing effectively complements DNA sequencing and increased the detection rate of gene fusions, supporting that NGS technology can effectively assist the diagnosis of bone tumors.

## Introduction

Bone tumor often refers to the tumor occurring in bone or its accessory tissues, including primary and metastatic (1). Compared with other tumors, bone tumors account for about 0.2% of all malignant tumors (2).The complexity and heterogeneity of bone tumors increase the difficulty of diagnosis and treatment (3). Osteosarcoma, chondrosarcoma, and Ewing’s sarcoma are common subtypes of bone tumors (4). Although surgical resection, radiotherapy, and chemotherapy have widely improved patient survival, bone tumors are still considered deadly in a high percentage of cases and seriously affect the quality of patients’ lives (5). Therefore, a further understanding of the mechanism of bone tumors for overcoming drug resistance and improving the low survival rate is still urgently needed.

With the development of molecular genetic technology, the molecular characteristics of bone tumors have been studied and some bone tumors can be diagnosed with the help of genomic mutations (6, 7). For example, Ewing’s sarcoma is characterized by chimeric fusion expression of EWS and ETS family transcription factors (6), Ewing-like sarcomas are characterized by the rearrangement of *CIC* and *BCOR* (8), giant cell tumor of bone is characterized by the mutation of *H3F3A* (p.G34W) (9), chondroblastoma is characterized by the mutation of *H3F3B* (p.K36M) (9), mesenchymal chondrosarcoma is characterized by the fusion of *HEY1-NCOA2* (10), chondromyxoid fibroma is characterized by *GRM1* rearrangements (11), aneurysmal bone cyst is characterized by *USP6* rearrangements (12), osteoblastoma/osteoid osteoma is characterized by *FOS/FOSB* rearrangements (13), and synovial chondromatosis is characterized by the fusion of *FN1-ACVR2A* and *ACVR2A-FN1* (14).

Osteosarcoma, the most common bone tumor, has begun to be a concern and related molecular characteristics have been studied. Previous studies show that the most frequent driver genes in osteosarcoma include *TP53*, *RB1*, *BRCA2*, *BAP1*, *RET*, *MUTYH*, *ATM*, *PTEN*, *WRN*, *RECQL4*, *ATRX*, *FANCA*, *NUMA1*, and *MDC1* (15, 16). Kovac et al. reported that more than 80% osteosarcomas have genomic mutational characteristic of *BRCA1*/2 deficient tumors (16). Behjati et al. elucidated mutational characterization and identified the mutations of insulin-like growth factor (IGF) signaling pathway in osteosarcomas by using whole-exome sequencing (WES) and whole-genome sequencing (WGS) based on a large cohort of 112 childhood and adult patients (17). In addition, aberrant signaling pathways, including PI3K/mTOR and Wnt signaling, were also reported in osteosarcomas (18). However, due to the low incidence and high heterogeneity, the efficacy of targeted therapy is still limited in osteosarcoma patients (19).

To date, the molecular mechanism of bone tumors, including osteosarcoma, chondrosarcoma, and Ewing’s sarcoma is still unclear. Although some bone tumors have been molecular typed, it is important to further elucidate and study the molecular characteristics of bone tumors for more accurate classification and treatment. In this study, we aimed to identify the molecular features of bone tumors and explore potential biomarkers for accurate diagnosis of bone tumor.

## Materials and Methods

### Ethics and statements

This study was approved by the Ethics Committee of the First Affiliated Hospital of Sun Yat-sen University (The approval number: [2018] 249). We declare that all methods used in this protocol were carried out in accordance with relevant guidelines and regulations. This study was approved by all patients and all participants provided informed consent.

### Patients and Sample Collection

A total of 357 bone tumor patients were enrolled in this study from First Affiliated Hospital of Sun Yat-sen University. Formalin-fixed paraffin-embedded (FFPE) tumor tissues and matched blood samples were collected. Genomic DNA was prepared by using QIAamp DNA/RNA FFPE Tissue Kit and QIAamp DNA Blood Midi Kit (Qiagen, Hilden, Germany) according to the manufacturer’s instructions. RNA was isolated by using miRNeasy FFPE Kit (Qiagen, Hilden, Germany) according to the manufacturer’s instructions. The concentration of DNA was measured by Qubit and normalized to 20–50 ng/μL for sequencing. Genomic alterations (GAs) were detected at Shanghai OrigiMed Co., Ltd (OrigiMed, Shanghai, China), a laboratory certified by College of American Pathologists (CAP) and Clinical Laboratory Improvement Amendments (CLIA).

### Identification of GAs and Tumor Mutational Burden (TMB)

The DNA/RNA samples were analyzed by using the next generation sequencing (NGS)-based YuanSu450 gene panel (OrigiMed, Shanghai, China), which covers all the coding exons of 450 tumor-related genes that are frequently rearranged in solid tumors. The genes were captured and sequenced with a mean depth of 800× by using Illumina NextSeq 500. The procedures followed the steps described by Frampton et al. (20). GAs identifications followed the previous published methods (21): Single nucleotide variants (SNVs) were identified by MuTect (v1.7). Insertion-deletions (Indels) were identified by using PINDEL (v0.2.5). The functional impact of GAs was annotated by SnpEff3.0. Copy number variation (CNV) regions were identified by Control-FREEC (v9.7) with the following parameters: window = 50 000 and step = 10 000. Gene fusion/rearrangements were assessed by Integrative Genomics Viewer (IGV). For RNA-Seq data, gene fusions were detected by using STAR-fusion (v1.4) (22). TMB was estimated by counting the coding somatic mutations, including SNVs and Indels, per megabase of the sequence examined in each patient. The TMB value was further divided into two groups: TMB-H, defined as ≥10 mutations/Mb, and TMB-L, defined as <10 mutations/Mb.

### Statistical Analysis

Statistical analyses were performed using SPSS version 22.0 (SPSS Inc., Chicago, IL, USA). Fisher’s exact test was used for the association analysis of categorical variables. Student’s t−test and Wilcoxon rank sum test were used for the association analysis of normally distributed data and non-normally distributed data, respectively. P < 0.05 was considered statistically significant.

## Results

### Clinical Characteristics of Bone Tumor Patients

The 357 bone tumor patients included 266 primary tumors, 50 metastatic tumors, 38 local recurrent tumors, and 3 tumors with unclear origin. There were 214 men and 143 women in this cohort and the median age was 20 years (range 1-86 years). The proportion of male patients is higher than the proportion of female patients in this cohort (60% *vs.* 40%). The proportion of primary tumors, metastatic tumors, and local recurrent tumors in male patients is higher than that in female patients (58% *vs.* 42%, 66% *vs.* 34%, and 61% *vs.*39%, respectively). According to pathology, this cohort includes 227 osteosarcomas, 43 chondrosarcomas, 14 chordomas, 13 giant cell tumors of bone, 12 malignant giant cell tumors of bone, 4 osteoblastomas, 4 undifferentiated sarcomas, 3 undifferentiated pleomorphic sarcomas, 1 low grade malignant fibromyxoid sarcomas, 1 myofibroblastomas, 1 chondromatosises, 1 fibrosarcomas, 1 hemangiomas, 1 angiosarcomas, and 31 unclassified bone tumors (**Table 1**).

**Table 1:** Clinicopathological features of Chinese bone tumor patients.

Subtypes	Gender		Age (Years)	TMB (muts/Mb)	Tumor Stage at presentation				Total
	Male	Female	(Median, range)	(Median, range)	Primary tumor	Local recurrence	Metastasis	Unknown	
Osteosarcoma	140	87	16 (4-76)	2.3 (0-52.7)	181	18	27	1	227
chondrosarcoma	21	22	46 (1-86)	2.3 (0-5.6)	31	6	6	0	43
chordoma	9	5	58.5 (35-69)	1.8 (0-3.9)	7	4	3	0	14
Giant cell tumor of bone	7	6	35 (14-54)	0.6 (0-1.8)	13	0	0	0	13
Malignant giant cell tumor of bone	5	7	31(20-55)	0.7 (0-5.7)	6	4	2	0	12
Osteoblastoma	3	1	12.5 (12-17)		4	0	0	0	4
undifferentiated sarcoma	3	1	48.5 (32-50)	1.4 (1.1-3.2)	3	1	0	0	4
undifferentiated pleomorphic sarcoma	2	1	43 (40-54)	3.1(1.5-6.9)	2	1	0	0	3
Low grade malignant fibromyxoid sarcoma	0	1	52	2.3	1	0	0	0	1
Myofibroblastoma	1	0	41	18.1	0	0	1	0	1
Chondromatosis	1	0	68	6.1	1	0	0	0	1
Fibrosarcoma	1	0	2	3	0	1	0	0	1
Hemangioma	0	1	54	0.7	1	0	0	0	1
Angiosarcoma	1	0	48	6.2	1	0	0	0	1
Unclear	20	11	23 (2-73)	1.8 (0-11.5)	15	3	11	2	31
Total	214	143	20 (1-86)	1.8 (0-52.7)	266	38	50	3	357

**Figure 1:**
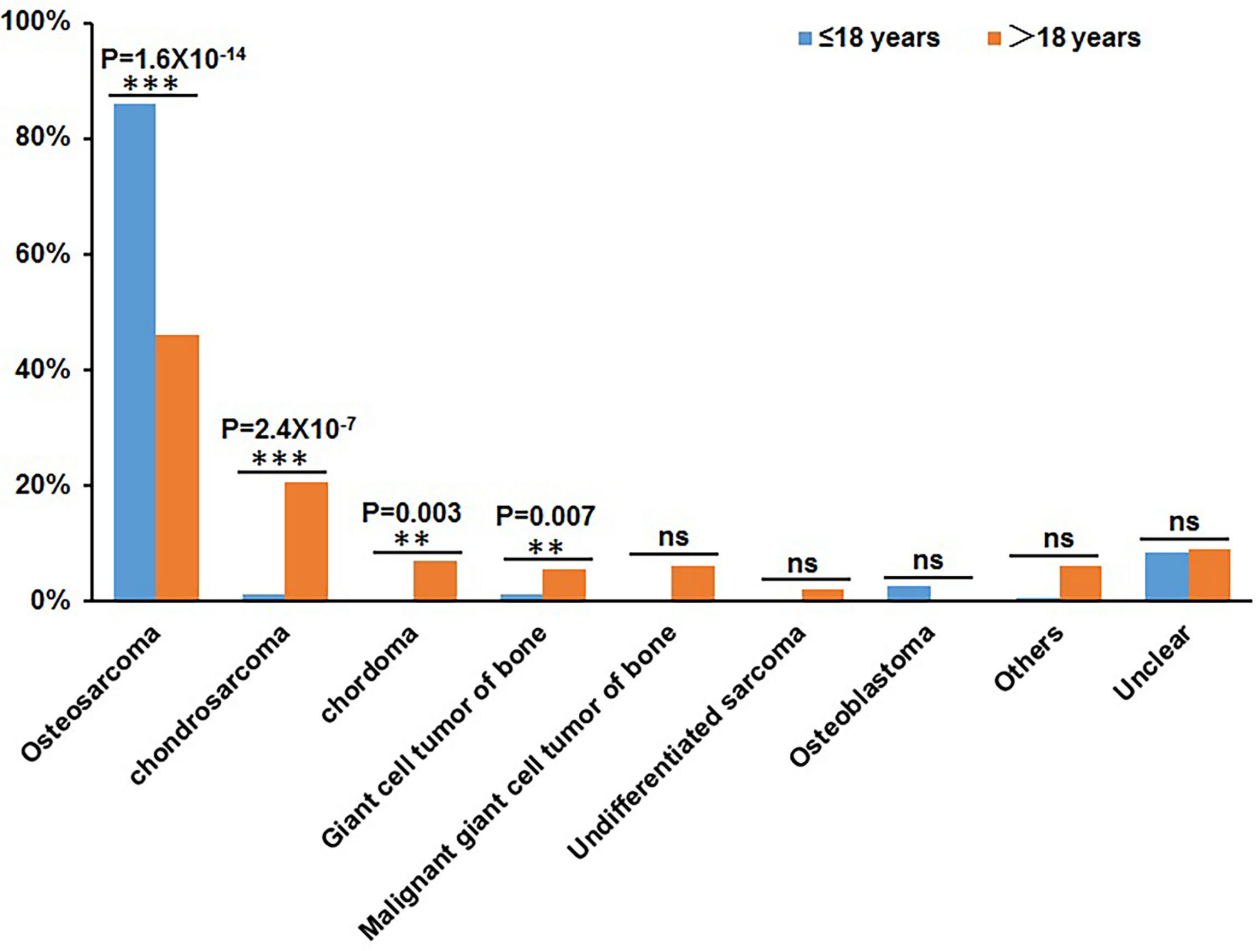
Statistical distribution of juvenile patients (≤18 years) and adult patients (>18 years) in bone tumor subtypes. The X-axis represents the different bone tumor subtypes and the Y-axis represents the proportion of juvenile patients and adult patients. **P < 0.01, ***P< 0.001, and “ns” represents no significant difference.

Patients over 18 years old were classified into adult groups and those under 18 years old (including 18 years old) were classified into juvenile group. There were 206 adult patients and 151 juvenile patients in this cohort. Based on the classification of bone tumor subtypes, we found that the proportions of osteosarcoma and osteoblastoma were higher in the juvenile group than that in adult group, but the proportions of chondrosarcoma, chordoma, giant cell tumors of bone, malignant giant cell tumors of bone, and undifferentiated sarcomas were lower in juvenile group than that in adult group. Statistical analysis shows the significant correlations between juvenile patients and osteosarcomas and between adult patients and chondrosarcoma (**Figure 1**). Meanwhile, statistical analysis shows that there is no difference between adult patients and juvenile patients regarding the distribution of tumor origin.

### GAs of Bone Tumor

**Figure 2:**
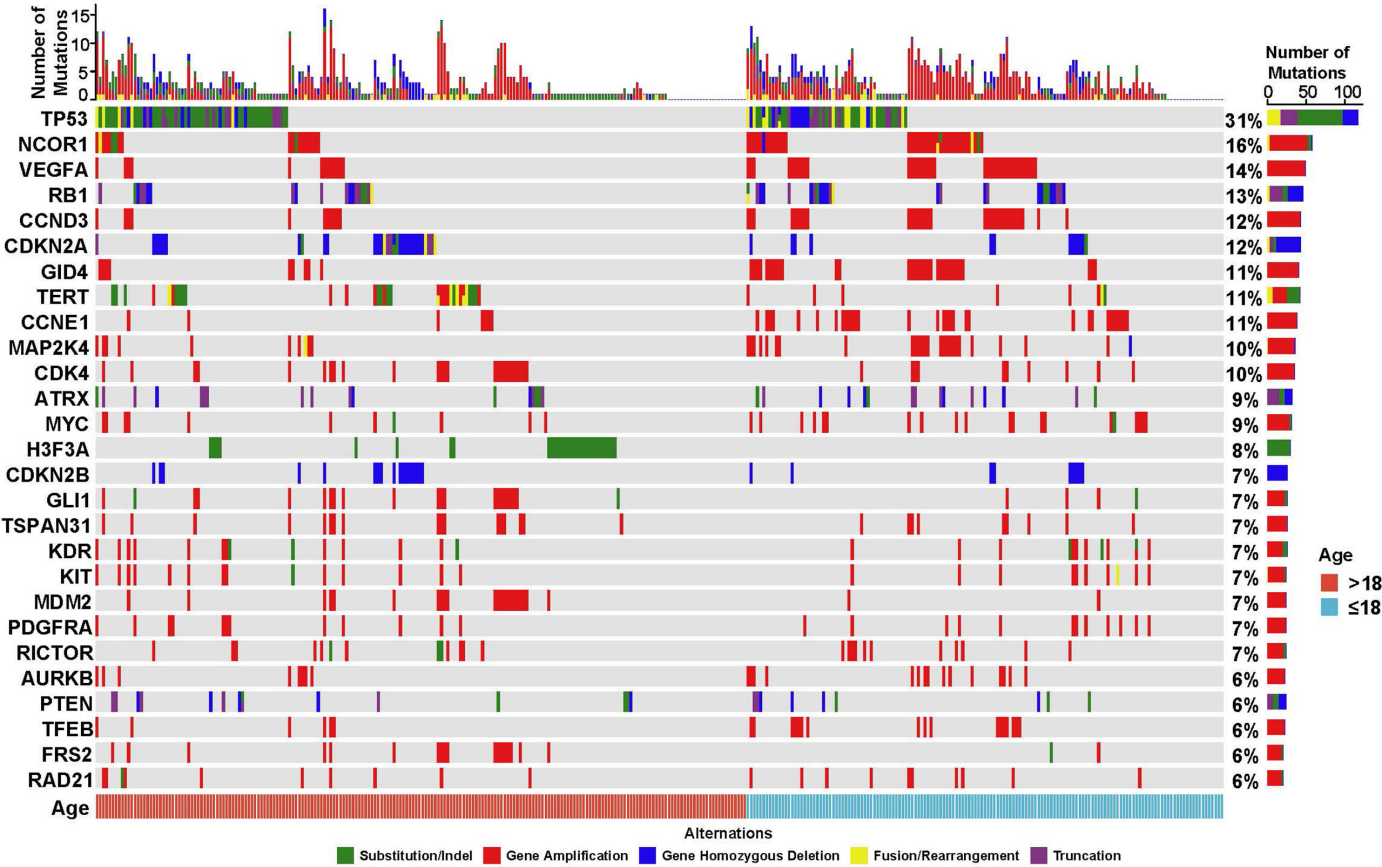
Mutational profiling of 357 Chinese bone tumor patients. The cohort was classified into juvenile (≤18 years, blue) and adult (>18 years, red) groups based on the age of patients. The X-axis represents each case sample and the Y-axis represents each mutated gene. The bar graph on the right shows the mutation number of each gene, and the bar graph above shows the mutation number of each sample. Green represents substitution/Indel mutations, red represents gene amplification mutations, blue represents gene homozygous deletion mutations, yellow represents fusion/rearrangement mutations, and purple represents truncation mutations.

A total of 2,780 clinically relevant GAs were identified (**Figure 2**), with average of 10.02 alterations per sample (ranged from 0 to 33). Among these GAs, gene amplification was the most frequent mutation type (57.01%, 1585/2780), followed by SNV/Short Indel (24.89%, 692/2780), gene homozygous deletion (6.15%, 171/2780), truncation (6.04%, 168/2780), and gene fusion/rearrangement (5.90%, 164/2780) (**Figure 2**; **Table S1**). The most commonly mutated genes with a mutation frequency of more than 10% were *TP53* (31.37%, 112/357), *NCOR1* (15.69%, 56/357), *VEGFA* (13.73%, 49/357), *RB1* (12.61%, 45/357), *CCND3* (12.04%, 43/357), *CDKN2A* (11.76%, 42/357), *GID4* (11.48%, 41/357), *TERT* (11.20%, 40/357), *CCNE1* (10.64%, 38/357), and *MAP2K4* (10.08%, 36/357).

In this cohort, germline mutations were detected in 28 patients, including 16 adult patients and 12 juvenile patients. The most common germline mutations include *MSH6*, *TP53*, *ATM*, *BRCA2*, and *FANCA* mutations. The mutations of *MSH6*, *TP53*, *ATM*, and *BRCA2* mainly occurred in adult patients, while the mutations of *FANCA* and *RAD51D* mainly occurred in juvenile patients. Thirteen (13) patients harbored homologous recombination deficiency (HRD) related mutations, including *ATM*, *FANCA*, *BRCA2*, *RAD50*, *RAD51D*, and *FANCD2*. A juvenile patient carried both *TP53* and *NBN* germline mutations (**Figure S1**).

### Potential Actionable Targeted Mutations in Bone Tumor Patients

Actionable targeted mutations are helpful for guiding targeted therapy and immunotherapy. In this cohort, a total of 145 actionable targeted mutations were identified in 122 (34.2%) bone tumor patients, including 81 osteosarcomas, 17 chondrosarcomas, 6 chordomas, 3 undifferentiated sarcomas, 2 undifferentiated pleomorphic sarcoma, 1 fibrosarcoma, 1 low grade fibromyxoid sarcoma, 1 osteoblastoma, and 10 bone tumors with unclear subtype (**Table 2**). Among these mutations, the most common actionable targeted mutations included *CDKN2A* mutation (34.4%, 42/122) targeted to Abemaciclib, Palbociclib, and Ribociclib, *PTEN* mutation (18.9%, 23/122) targeted to GSK2636771 and AZD8186, *NF1* mutation (13.9%, 17/122) targeted to Trametinib, Cobimetinib, and *FGFR1* mutation (11.5%, 11/122) targeted to AZD4547, Debio1347, Infigratinib, and Erdafitinib (**Table 2**; **Figure S2**). Due to the high percentage of osteosarcomas, nearly 65% of actionable targeted mutations were detected in osteosarcomas.

**Table 2:** Actionable targeted mutations in this cohort.

Actionable targeted mutations	Case number	Mutational frequency of actionable mutated genes (n/122)	Targeted drug
*CDKN2A*	42	34.4%	Abemaciclib, Palbociclib, Ribociclib
*PTEN*	23	18.9%	GSK2636771, AZD8186
*NF1*	17	13.9%	Trametinib, Cobimetinib
*FGFR1*	14	11.4%	AZD4547, Debio1347, Infigratinib, Erdafitinib
*KRAS*	11	9.0%	Binimetinib, Trametinib, Cobimetinib
*NTRK3*	9	7.4%	Entrectinib
*ARID1A*	5	4.1%	PLX2853
*BRAF*	5	4.1%	PLX8394
*FGFR2*	4	3.3%	Infigratinib, AZD4547, Debio1347, Erdafitinib
*FGFR3*	4	3.3%	Erdafitinib, Infigratinib, Debio1347, AZD4547
*MET*	4	3.3%	Crizotinib
*MTOR*	2	1.6%	Temsirolimus, Everolimus
*NTRK1*	2	1.6%	Entrectinib
*NTRK2*	2	1.6%	Entrectinib
*CDK12*	1	0.8%	Pembrolizumab, Cemiplimab, Nivolumab
Total	145		

### RNA Sequencing Effectively Increases the Detection Rate of Gene Fusions in Bone Tumor Patients

A total of 179 gene fusions were detected in 130 bone tumor patients. Among them, 3 harbored 4 different gene fusions, 7 harbored 3 different gene fusions, and 26 harbored 2 different gene fusions. The most common gene fusion was *TP53*, followed by *TERT*, *NTRK3*, *ETV6*, *NF1*, *KMT2D*, and *LRP1* (**Table S1**). Most of the fusions occurred on one chromosome and chromosome 17 and chromosome 12 were the chromosomes on which the most frequent fusions occurred. The fusions between chromosomes commonly occurred between chromosomes 6 and 17, chromosomes 2 and 12, and chromosomes 14 and 17. Chromosome 10 tended to fuse with other chromosomes rather than with itself. Most of the fusions on chromosome 10 belonged to the fusions between chromosomes (**Figure S3**).

Notably, GAs in 191 patients were detected by DNA and RNA sequencing and 113 gene fusions were detected in 79 patients (41.4%, 79/191). Nineteen (16.8%, 19/113) gene fusions such as *SH2D2A*-*NTRK1*, *EMILIN1*-*ALK*, *ETV6*-*CNTN1*, and *SZRD1*-*SPEN*, were detected in 16 (20%, 16/79) patients with the assistance of RNA sequencing (**Table 3**).

**Table 3:** Gene fusions detected by the assistance of RNA sequencing.

Order	GENE_PAIR A	GENE_L A	GENE_PAIR B	GENE_L B
1	*NRP2*	chr2	*FYN*	chr6
	*ICK*	chr6	*KIT*	chr4
2	*INSL3*	chr19	*JAK3*	chr19
3	*RAD52*	chr12	*PLCB1*	chr20
4	*KMT2D*	chr12	*PRKAG1*	chr12
5	*LMF1*	chr16	*CREBBP*	chr16
	*RECQL5*	chr17	*GRIN2A*	chr16
6	*ETV6*	chr12	*CNTN1*	chr12
7	*PRKG1*	chr10	*ETV1*	chr7
8	*FGFR1*	chr8	*SERPINB7*	chr18
9	*EMILIN1*	chr2	*ALK*	chr2
10	*NRP1*	chr10	*TERT*	chr5
11	*SEC16A*	chr9	*NOTCH1*	chr9
12	*FAM131C*	chr1	*EPHA2*	chr1
13	*COL5A1*	chr9	*RXRA*	chr9
14	*SZRD1*	chr1	*SPEN*	chr1
15	*ABL1*	chr9	*FUBP3*	chr9
	*SLIT2*	chr4	*MBD5*	chr2
16	*SH2D2A*	chr1	*NTRK1*	chr1

### Age May Be a Key Clinical Factor in the Treatment of Bone Tumors

Considering the different tendencies of adult and juvenile patients in different bone tumor subtype, we further analyzed the mutated genes in each patients group. The most frequent mutated genes in adult patients group included *TP53* (29.61%, 61/206), *TERT* (15.53%, 32/206), *CDKN2A* (14.56%, 30/206), *CDK4* (12.14%, 25/206), *MDM2* (10.68%, 22/206), and *GLI1* (10.68%, 22/206). While the most common mutated genes included *TP53* (33.77%, 51/151), *NCOR1* (24.50%, 37/151), *VEGFA* (23.84%, 36/151), *CCND3* and *GID4* (21.19%, 32/151, for both), *CCNE1* (20.53%, 31/151), *RB1* and *MAP2K4* (17.22%, 26/151, for both), *MYC* (13.25%, 20/151), and *TFEB* (11.26%, 17/151) in juvenile patients. Statistical analysis showed that the mutations of *NCOR1* (P=0.021), *VEGFA* (P=0.0012), *CCND3* (P=0.0028), *GID4* (P=6.0X10^-4^), *CCNE1* (P=3.4X10^-4^), *RB1* (P=0.037), and *MAP2K4* (P=0.025) were significantly associated with the juvenile patient group, while the mutation of *H3F3A* (P=2.5X10^-6^) was significantly associated with the adult patient group (**Table 4**). In addition to the mutations of *RB1* and *MAP2K4*, most mutations of *NCOR1*, *VEGFA*, *CCND3*, *GID4*, and *CCNE1* were gene amplification (**Figure 2**).

**Table 4:** The most common mutated genes and their distribution in adult patients and juvenile patients.

GENE	Adult proportion	Juvenile proportioin	P-value
*TP53*	34.47%	45.70%	0.47
*TERT*	16.50%	5.96%	0.19
*CDKN2A*	15.53%	7.95%	0.08
*H3F3A*	14.56%	0.00%	^***^2.5X10^-6^
*CDK4*	12.62%	6.62%	0.12
*GLI1*	11.17%	2.65%	0.25
*MDM2*	11.17%	1.32%	0.052
*NCOR1*	10.68%	26.49%	^*^0.021
*RB1*	10.19%	18.54%	^*^0.037
*VEGFA*	6.31%	24.50%	^**^0.0012
*CCND3*	5.34%	21.85%	^**^0.0028
*GID4*	4.37%	21.19%	^***^6.0X10^-4^
*CCNE1*	3.40%	20.53%	^***^3.4X10^-4^
*MAP2K4*	5.34%	17.88%	^*^0.025
*MYC*	5.34%	13.25%	0.66
*TFEB*	2.91%	11.26%	0.19
*AURKB*	4.37%	10.60%	0.65

*P < 0.05, **P < 0.01, and ***P < 0.001.

### Significant Mutational Characteristics of Osteosarcoma Patients

**Figure 3:**
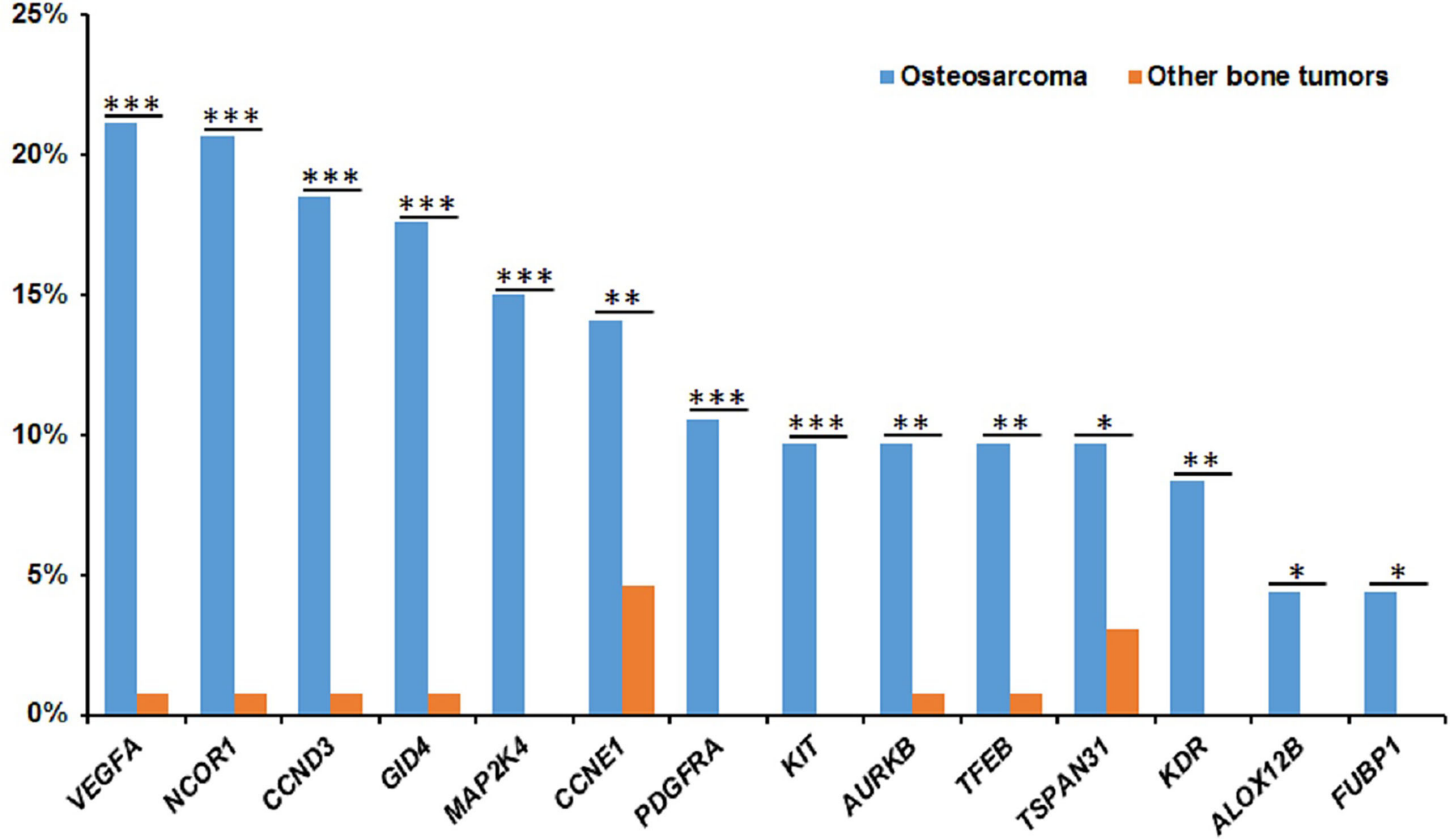
The significantly frequent gene amplifications in osteosarcomas. The X-axis shows the genes and the Y-axis shows the proportions of gene amplifications in osteosarcomas or other bone tumors. Blue represent osteosarcomas and red represent other bone tumors. *P < 0.05, **P < 0.01, and ***P< 0.001.

Except for osteosarcoma, the patient number of other bone tumor subtypes is so small that the results of separate analysis are not representative. Therefore, we especially characterized the molecular profiling of osteosarcoma. Similar to the whole bone tumor cohort, the most common mutated genes in osteosarcoma included *TP53*, *NCOR1*, *VEGFA*, *CCND3*, *GID4*, *RB1*, *MAP2K4*, *CCNE1*, *ATRX*, *CDK4*, *CDKN2A* , *MYC*, and *PDGFRA* (**Figure S4**). In osteosarcoma, the most common mutation was gene amplification, which accounts for about 84% (1336/1591) of the whole bone tumor cohort. The most frequently amplified gene was *VEGFA* (21.1%, 48/227), followed by NCOR1 (20.7%, 47/227), *CCND3* (18.5%, 42/227), *GID4* (17.6%, 40/227), *MAP2K4* (15.0%, 34/227), *CCNE1* (14.1%, 32/227), *CDK4* (12.3%, 28/227), *PDGFRA* (10.6%, 24/227), and *MYC* (10.1%, 23/227). Compared with other bone tumor subtypes, the amplification of *NCOR1* (P=2.6x10^-7^), *VEGFA* (P=1.8x10^-7^), *CCND3* (P=1.7x10^-6^), *GID4* (P=3.6x10^-6^), *MAP2K4* (P=8.5x10^-6^), *CCNE1*(P=8.9x10^-3^), *AURKB* (P=2.1x10^-3^), *PDGFRA* (P=3.0x10^-4^), *KIT* (P=5.9x10^-4^), *TSPAN31* (P=0.035), *TFFB* (P=2.1x10^-3^), *KDR* (P=1.7x10^-3^), *ALOX12B* (P=0.036), and *FUBP1* (P=0.036) were more frequent in osteosarcoma (**Figure 3**). Interestingly, the copy number of *NCOR1* (P=0.04) in juvenile patients was significantly more than that in adult patients.

### Application of NGS in the Diagnosis of Bone Tumors

**Figure 4:**
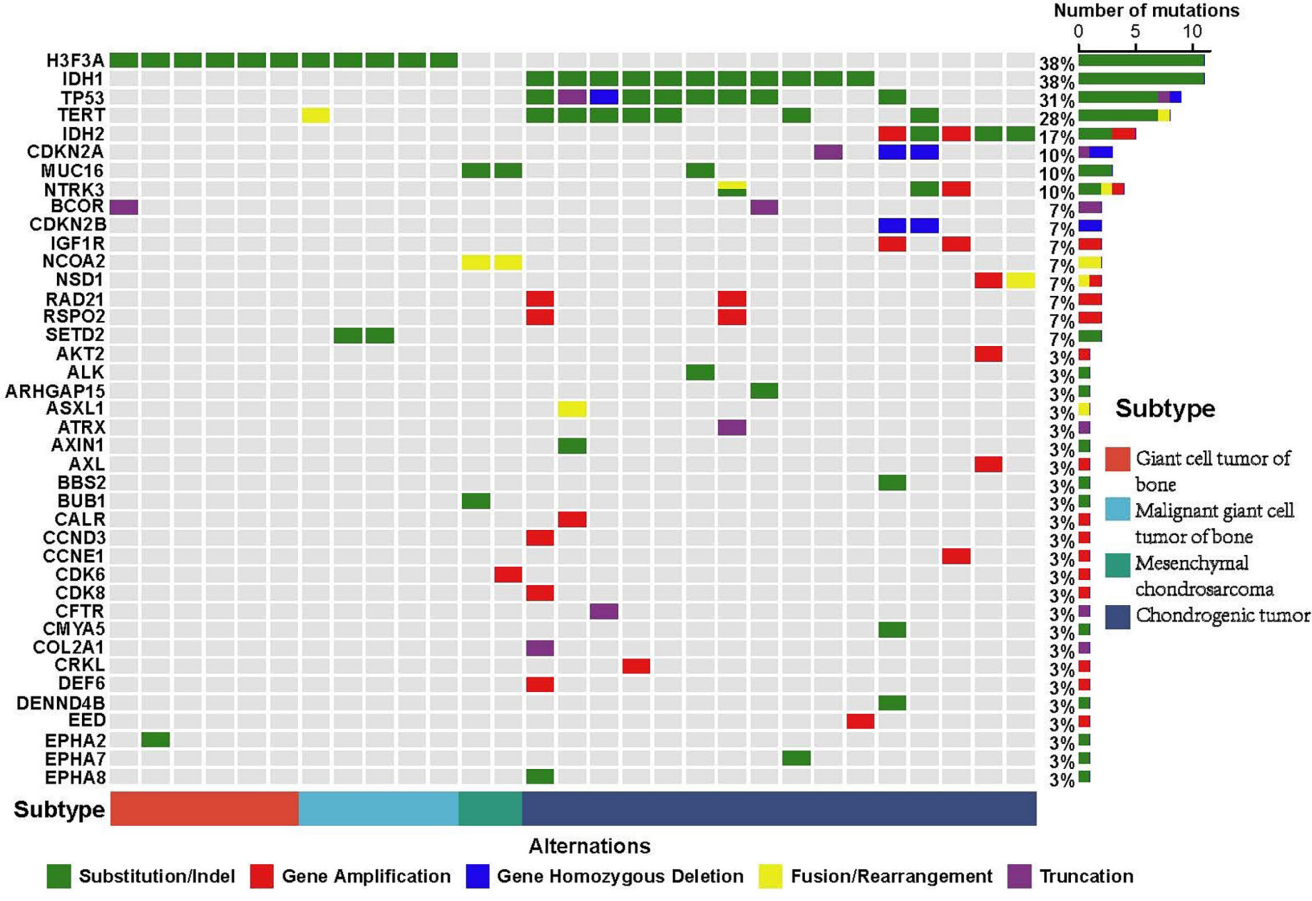
Mutational characterization of confirmed subtypes of bone tumors. The well-known bone tumor subtype includes giant cell tumor of bone (red), malignant giant cell tumor of bone (blue), mesenchymal chondrosarcoma (green), and chondrogenic tumor (dark blue). The X-axis represents each case sample and the Y-axis represents each mutated gene. The bar graph on the right shows the mutation number of each gene and the bar graph above shows the mutation number of each sample. Green represents substitution/Indel mutations, red represents gene amplification mutations, blue represents gene homozygous deletion mutations, yellow represents fusion/rearrangement mutations, and purple represents truncation mutations.

According to the WHO classification of bone tumors (fifth edition), many subtypes including giant cell tumor of bone, malignant giant cell tumor of bone, and chondrosarcoma can be classified on the basis of their molecular characterization. According to the confirmed subtypes of bone tumors, we also investigated the alterations of giant cell tumor of bone, malignant giant cell tumor of bone, mesenchymal chondrosarcoma, and chondrogenic tumor in this cohort. Results showed that mutations of H3F3A were detected in all giant cell tumor of bone and malignant giant cell tumor of bone, and mutations of *IDH1/2* (including 10 *IDH1* and 5 *IDH2*) were detected in chondrogenic tumors. Also, the fusion of *HEY1*-*NCOA2* and mutations of *MUC16* were detected in all mesenchymal chondrosarcomas (**Figure 4**).

There are many subtypes of bone tumors and, at times, the precise diagnosis can be a challenge. NGS detection is an effective strategy for auxiliary diagnosis. As an example: pathological examination of a female patient showed that a large number of flaky distributed epithelioid or oval spindle cells with high anaplastic, pleomorphic characteristics, and mitosis was easy to be observed, along with a locally distributed bone like matrix and multinucleated giant cells (**Figure S5**). These pathological features do not exclude the possibility of both osteosarcoma and giant cell tumor/malignant giant cell tumor of bone. Based on a mutation of *H3F3A* G35W detected using NGS technology, this case was finally diagnosed as a giant cell tumor/malignant giant cell tumor of bone.

In addition to this case, we also confirmed the initial diagnosis of bone tumors by using NGS detection. In total, the diagnoses of 26 (7.28%, 26/357) bone tumors including 15 osteosarcomas, 6 chondrosarcomas, 1 fibrosarcoma of bone, 1 malignant giant cell tumor of bone, 1 spindle cell tumor, 1 undifferentiated sarcoma, and 1 unclassified bone tumor were modified with the assistance of NGS technology. Based on the results of NGS detection, 12 cases harbored the mutation of *H3F3A G35W/L/R* which supported the diagnosis of giant cell/malignant giant cell tumors of bone; 6 cases harbored the mutation of *GNAS R201H/C* which supported the diagnosis of fibrous dysplasia of bone; 2 cases harbored *NTRK3*-intergenic and *NTRK3-UQCRC1*, respectively, which supported the diagnosis of NTRK rearranged spindle cell tumors; 2 cases harbored *IDH1 R132C* which supported the diagnosis of endophytic chondroma; 1 case harbored *BCOR-CCNB3* fusion which supported the diagnosis of *BCOR-CCNB3* featured sarcoma; 1 case harbored *EWSR1-NR4A3* fusion which supported the diagnosis of extraskeletal myxoid chondrosarcoma; 1 case harbored *SS18-SSX1* fusion which supported the diagnose of synovial sarcoma; and 1 case harbored *MDM2* and *CDK4* amplifications which supported the diagnosis of low grade paraosseous/intraosseous osteosarcoma (**Table 5**).

**Table 5:** The list of auxiliary diagnosed cases.

ORDER	Primary diagnosis	Auxiliary diagnosis	Mutation characteristics
1	Osteosarcomas	Malignant giant cell tumor of bone	*H3F3A G35W*
2	Osteosarcomas	Malignant giant cell tumor of bone	*H3F3A G35W*
3	Osteosarcomas	Malignant giant cell tumor of bone	*H3F3A G35W*
4	Osteosarcomas	Malignant giant cell tumor of bone	*H3F3A G35W*
5	Osteosarcomas	Malignant giant cell tumor of bone	*H3F3A G35W*
6	Osteosarcomas	Malignant giant cell tumor of bone	*H3F3A G35L*
7	Chondrosarcomas	Malignant giant cell tumor of bone	*H3F3A G35W*
8	Undifferentiated sarcoma	Malignant giant cell tumor of bone	*H3F3A G35L*
9	Fibrosarcoma of bone	Malignant giant cell tumor of bone	*H3F3A G35W*
10	Osteosarcomas	Malignant giant cell tumor of bone/giant cell tumor of bone	*H3F3A G35W*
11	Osteosarcomas	Giant cell tumor of bone	*H3F3A G35W*
12	Osteosarcomas	Giant cell tumor of bone	*H3F3A G35R*
13	Osteosarcomas	Fibrous dysplasia of bone	*GNAS R201H*
14	Malignant giant cell tumor of bone	Fibrous dysplasia of bone	*GNAS R201H*
15	Osteosarcomas	Fibrous dysplasia of bone	*GNAS R201H*
16	Osteosarcomas	Fibrous dysplasia of bone	*GNAS R201H*
17	Spindle cell tumor	Fibrous dysplasia of bone	*GNAS R201C*
18	Unclassified	Fibrous dysplasia of bone	*GNAS R201C*
19	Osteosarcomas	*BCOR-CCNB3* featured sarcoma	*BCOR-CCNB3*
20	Osteosarcomas	NTRK rearranged spindle cell tumors	*NTRK3-intergenic*
21	Osteosarcomas	NTRK rearranged spindle cell tumors	*NTRK3-UQCRC1*
22	Chondrosarcomas	Extraskeletal myxoid chondrosarcoma	*EWSR1-NR4A3*
23	Chondrosarcomas	Synovial sarcoma	*SS18-SSX1*
24	Chondrosarcomas	Endophytic chondroma	*IDH1 R132C*
25	Chondrosarcomas	Endophytic chondroma	*IDH1 R132C*
26	Chondrosarcomas	Low grade paraosseous/intraosseous osteosarcoma	*CDK4* and *MDM2* Amplification

## Discussion

Bone tumors occur widely in children and adolescents and face the problems of difficult treatment and high mortality (23). Bone tumors include many subtypes such as osteosarcoma, chondrosarcoma, chordomas, giant cell tumors of bone, and osteoblastomas (3, 4). However, due to the rarity of bone tumors and the small number of patients, the molecular mechanism of bone tumors is still unclear. While genome sequencing research of bone tumors are underway, small sample number and limited bone tumor subtypes are the common deficiencies (17, 24, 25). In this study, we investigated the genomic alterations of 357 bone tumors with 16 subtypes, and osteosarcoma and chondrosarcoma were the main subtypes. Benign bone tumors are easy to cure and have a good prognosis, while malignant bone tumors have a poor prognosis and are easy to relapse. Therefore, the differential diagnosis of benign and malignant tumors is one key step in the diagnosis and treatment. Understanding the molecular characteristics of bone tumors is helpful for differential diagnosis, which is of great significance to guide patients’ treatment and improve patients’ prognosis.

NGS studies have been widely performed in osteosarcomas in Western countries. Previous studies showed that *TP53*, *RB1*, and *CCND3* frequently occurred in osteosarcoma (15, 26, 27). Notably, we identified the highly frequent *NCOR1* and *VEGFA* amplifications in our cohort, and the significantly higher *NCOR1* copy number in juvenile patients than that in adult patients.

*NCOR*1 is a nuclear receptor co-repressor and mediates transcriptional repression by certain nuclear receptors (28). The mutations in the *NCOR1* were reported to be associated with the prognosis of hormone receptor negative breast and lung adenocarcinoma (29). Yan et al. reported that *NCOR1* amplification frequently occurred in high-grade osteosarcoma (30). Selvarajah et al. showed that NCOR1 is negatively correlated with the prognosis of osteosarcoma in canines (31). These results suggest that *NCOR1* amplification may be a potential biomarker in osteosarcoma.

Inhibition of vascular endothelial growth factor (VEGF) signaling may lead to tumor-induced angiogenesis and inhibition of tumor growth (32, 33). In osteosarcoma, Yang et al. first reported the vascular endothelial growth factor A (*VEGFA*) gene amplification in osteosarcoma patients from Tianjin, China, and pointed that increasing *VEGFA* expression is the biomarker for poor prognosis of Chinese osteosarcoma (34). Combined with our study, we deduced that there may be different molecular mechanisms between osteosarcoma patients in China and those in western countries. The high proportion of *NCOR1* and *VEGFA* amplification may be a special molecular feature of Chinese osteosarcoma patients. Age is an important factor associated with the incidence of bone tumors. It is reported that the most of osteosarcomas occur in adolescents and chondrosarcoma usually occur in adults patients (35). Similarly, our results also showed the high incidence of osteosarcoma and chondrosarcoma in juveniles and adults patients, respectively. Also, we found the high incidence of osteoblastoma, but low incidence of chordoma, giant cell tumors of bone, malignant giant cell tumors of bone, and undifferentiated pleomorphic sarcomas in juvenile patients. The achieved results supplement the understanding of the incidence inclination of bone tumor subtypes in different ages.

Based on the statistical analysis, we identified a series of mutated genes that were correlated with juvenile/adult patients group. Notably, most of juvenile related mutations were also significantly associated with the osteosarcoma. The largest number of osteosarcoma patients in this cohort is a potential reason for this. However, these results also support that osteosarcoma mainly occurred in the juvenile patient group.

Due to the complexity and heterogeneity, a clear classification of bone tumors is often challenging. Significant progress has been made in the genotyping of bone tumors. For example, *IDH1*/*2* mutations are the molecular feature to distinguish chondrosarcoma from osteosarcoma (36), the high frequency of *MDM2* and *CDK4* amplifications in low-grade osteosarcoma (37), the high frequency of *NCOA2* fusion in mesenchymal chondrosarcoma (38), and the high frequency of *H3F3A* and *H3F3B* mutations in giant cell tumor of bone and chondroblastoma, respectively (39, 40). In this cohort, we also modified the diagnosis of 20 bone tumors based on the results of NGS detection, including 6 fibrous dysplasia of bone modified from 3 osteosarcomas, 1 malignant giant cell tumor of bone, 1 spindle cell tumor, and 1 unclassified bone tumor. These results supported that NGS-based assisted diagnosis is of great significance to improve the benefit to patients.

In addition, the rapid development of NGS technology in the past decades has led to the discovery of new tumor mutation features and more accurate differential diagnosis. The 5^th^ edition of the WHO classification has named some soft tissue sarcomas or bone tumors by newly recognized molecular and genetic alterations (41). According to the primary diagnosis in this cohort, we also detected *H3F3A* in giant cell tumor of bone and malignant giant cell tumor of bone, *IDH1/IDH2* mutations in chondrosarcoma, and *NCOA2* fusion in mesenchymal chondrosarcoma. Notably, we also found *MUC16* mutations in these tumors. Muc16 is a transmembrane protein recognized by monoclonal antibody CA125 (42). Li et al. reported that the *MUC16* mutation may be associated with tumor mutational burden and outcomes in gastric adenocarcinoma patients (43). Based on a large cohort of 10,195 patients, the *MUC16* mutation was shown to be associated with response to, and improved outcomes for, ICI treatment in solid tumors (44). Although there are only two cases, considering the rarity of mesenchymal chondrosarcoma, the identification of the *MUC16* mutation indicates the potential opportunity of mesenchymal chondrosarcoma patients to benefit from immunotherapy. Targeted therapy for bone tumors is rarely reported. In this study, it was found that about 34% of Chinese bone tumor patients harbored actionable targeted mutations, including *CDKN2A* , *PTEN*, *FGFR1/2/3*, *NF1*, and *NTRK1/2/3*. Unfortunately, except for targeting a few gene fusions such as *NTRK1/2/3*, targeted therapy for bone tumors has made slow progress. Gene fusions are a common genomic variation in tumors and especially in bone tumors, oncogenic gene fusions commonly occur (45). Chromoplexy is the main cause of gene fusion generated in bone and soft tissue tumors, including gene fusions within and between chromosomes (46). In this study, our results showed that gene fusion within chromosomes mainly occurred in bone tumors, and the gene fusion is inclined to occur on chromosome 17 and chromosome 12, including the fusion of *TP53*, *ETV6*, and *KMT2D*. Although Ewing’s sarcoma is not considered to be included in osteosarcoma (41), the identification of fusion in osteosarcoma is still useful for auxiliary diagnosis. However, it is inevitable that gene fusion will missed due to the concentration and abundance of DNA or RNA. In this study, our results confirmed that the combination of DNA and RNA detection can effectively increase the detection rate of gene fusion. This is of great significance in the auxiliary diagnosis and subsequent treatment of osteosarcoma and sarcoma.

In conclusion, we identified the mutation characteristics of bone tumor patients, pointed out the differences of germline and somatic mutations between juvenile patients and adult patients, and increased the efficiency of fusion detection by the combination of DNA and RNA sequencing. Our results supported that NGS technology can effectively assist in the diagnosis of bone tumors and provided the evidence for the precise treatment of bone tumors.

## Data Availability Statement

The original contributions presented in the study are included in the article/**Supplementary Material**. Further inquiries can be directed to the corresponding author.

## Ethics Statement

The studies involving human participants were reviewed and approved by Ethics Committee of The First Affiliated Hospital of Sun Yat-sen University. The patients/participants provided their written informed consent to participate in this study. Written informed consent was obtained from the individual(s) for the publication of any potentially identifiable images or data included in this article.

## Author Contributions

XX, YB, HL, JY, LT, RJ, ZZ, ZL, and CH collected patient consents and samples and analyzed data; XX, YQ, XS, and LW contributed to bioinformatics analysis and wrote the manuscript; JS designed and supervised the study. All authors read and approved the final manuscript.

## Funding

This study was supported by grants from the National Natural Science Foundation of China (No. 81972510 and No. 81772861).

## Conflict of Interest

Authors XS, YQ, and LW were employed by Shanghai OrigiMed Co., Ltd.

The remaining authors declare that the research was conducted in the absence of any commercial or financial relationships that could be construed as a potential conflict of interest.

## Publisher’s Note

All claims expressed in this article are solely those of the authors and do not necessarily represent those of their affiliated organizations, or those of the publisher, the editors and the reviewers. Any product that may be evaluated in this article, or claim that may be made by its manufacturer, is not guaranteed or endorsed by the publisher.
