# Supplementary material for: A Comprehensive Understanding of the Genomic Bone Tumor Landscape: A Multicenter Prospective Study
Source: Front Oncol. 2022 Jun 8;12:835004. doi: 10.3389/fonc.2022.835004 (PMC9213736; doi:10.3389/fonc.2022.835004)
Supplement: Supplementary file 1 [file Table_1.docx]

Table S1 Total genomic alterations detected in different genes.

| Genes | Gene Amplification | Substitution/Indel | Gene Homozygous Deletion | Truncation | Fusion/Rearrangement | Total | Ratio |
| --- | --- | --- | --- | --- | --- | --- | --- |
| TP53 | 0 | 56 | 18 | 23 | 15 | 112 | 31.37% |
| NCOR1 | 48 | 5 | 1 | 0 | 2 | 56 | 15.69% |
| VEGFA | 49 | 0 | 0 | 0 | 0 | 49 | 13.73% |
| RB1 | 0 | 6 | 19 | 17 | 3 | 45 | 12.61% |
| CCND3 | 43 | 0 | 0 | 0 | 0 | 43 | 12.04% |
| CDKN2A | 0 | 4 | 30 | 5 | 3 | 42 | 11.76% |
| GID4 | 41 | 0 | 0 | 0 | 0 | 41 | 11.48% |
| TERT | 19 | 16 | 0 | 0 | 5 | 40 | 11.20% |
| CCNE1 | 38 | 0 | 0 | 0 | 0 | 38 | 10.64% |
| MAP2K4 | 34 | 0 | 1 | 0 | 1 | 36 | 10.08% |
| CDK4 | 35 | 0 | 0 | 0 | 0 | 35 | 9.80% |
| ATRX | 0 | 7 | 9 | 16 | 0 | 32 | 8.96% |
| MYC | 29 | 2 | 0 | 0 | 0 | 31 | 8.68% |
| H3F3A | 0 | 30 | 0 | 0 | 0 | 30 | 8.40% |
| CDKN2B | 0 | 0 | 26 | 0 | 0 | 26 | 7.28% |
| GLI1 | 23 | 3 | 0 | 0 | 0 | 26 | 7.28% |
| TSPAN31 | 26 | 0 | 0 | 0 | 0 | 26 | 7.28% |
| KDR | 19 | 6 | 0 | 0 | 0 | 25 | 7.00% |
| KIT | 22 | 1 | 0 | 0 | 1 | 24 | 6.72% |
| MDM2 | 24 | 0 | 0 | 0 | 0 | 24 | 6.72% |
| PDGFRA | 24 | 0 | 0 | 0 | 0 | 24 | 6.72% |
| RICTOR | 21 | 3 | 0 | 0 | 0 | 24 | 6.72% |
| AURKB | 23 | 0 | 0 | 0 | 0 | 23 | 6.44% |
| PTEN | 0 | 6 | 9 | 8 | 0 | 23 | 6.44% |
| TFEB | 23 | 0 | 0 | 0 | 0 | 23 | 6.44% |
| FRS2 | 19 | 1 | 0 | 0 | 0 | 20 | 5.60% |
| RAD21 | 19 | 1 | 0 | 0 | 0 | 20 | 5.60% |
| ARHGEF25 | 19 | 0 | 0 | 0 | 0 | 19 | 5.32% |
| HMGA2 | 19 | 0 | 0 | 0 | 0 | 19 | 5.32% |
| KDM5A | 17 | 1 | 0 | 1 | 0 | 19 | 5.32% |
| CALR | 18 | 0 | 0 | 0 | 0 | 18 | 5.04% |
| IGF1R | 15 | 3 | 0 | 0 | 0 | 18 | 5.04% |
| NF1 | 0 | 4 | 5 | 4 | 4 | 17 | 4.76% |
| LRP1B | 0 | 9 | 5 | 1 | 1 | 16 | 4.48% |
| MTAP | 0 | 0 | 15 | 0 | 1 | 16 | 4.48% |
| RAD52 | 15 | 0 | 0 | 0 | 1 | 16 | 4.48% |
| MUC16 | 2 | 13 | 0 | 0 | 0 | 15 | 4.20% |
| COL1A1 | 5 | 3 | 0 | 5 | 1 | 14 | 3.92% |
| FGFR1 | 8 | 4 | 0 | 0 | 2 | 14 | 3.92% |
| GNAS | 8 | 6 | 0 | 0 | 0 | 14 | 3.92% |
| KMT2D | 0 | 5 | 0 | 5 | 4 | 14 | 3.92% |
| LRP1 | 3 | 7 | 0 | 0 | 4 | 14 | 3.92% |
| MCL1 | 14 | 0 | 0 | 0 | 0 | 14 | 3.92% |
| PIM1 | 14 | 0 | 0 | 0 | 0 | 14 | 3.92% |
| FAM135B | 6 | 7 | 0 | 0 | 0 | 13 | 3.64% |
| IL7R | 13 | 0 | 0 | 0 | 0 | 13 | 3.64% |
| RAC1 | 12 | 1 | 0 | 0 | 0 | 13 | 3.64% |
| BCOR | 3 | 1 | 0 | 7 | 1 | 12 | 3.36% |
| FOS | 8 | 1 | 0 | 0 | 3 | 12 | 3.36% |
| RSPO2 | 10 | 2 | 0 | 0 | 0 | 12 | 3.36% |
| SKP2 | 11 | 1 | 0 | 0 | 0 | 12 | 3.36% |
| ALOX12B | 10 | 1 | 0 | 0 | 0 | 11 | 3.08% |
| CCND2 | 11 | 0 | 0 | 0 | 0 | 11 | 3.08% |
| HDAC9 | 10 | 1 | 0 | 0 | 0 | 11 | 3.08% |
| IDH1 | 0 | 11 | 0 | 0 | 0 | 11 | 3.08% |
| JAK1 | 9 | 0 | 0 | 1 | 1 | 11 | 3.08% |
| KRAS | 9 | 2 | 0 | 0 | 0 | 11 | 3.08% |
| SDHA | 8 | 0 | 1 | 0 | 2 | 11 | 3.08% |
| CARD11 | 7 | 3 | 0 | 0 | 0 | 10 | 2.80% |
| FGF23 | 10 | 0 | 0 | 0 | 0 | 10 | 2.80% |
| FGF6 | 9 | 1 | 0 | 0 | 0 | 10 | 2.80% |
| FOXP1 | 2 | 3 | 0 | 3 | 2 | 10 | 2.80% |
| FUBP1 | 10 | 0 | 0 | 0 | 0 | 10 | 2.80% |
| GNA13 | 9 | 1 | 0 | 0 | 0 | 10 | 2.80% |
| JAK2 | 8 | 2 | 0 | 0 | 0 | 10 | 2.80% |
| KDM6A | 6 | 1 | 2 | 0 | 1 | 10 | 2.80% |
| KMT2C | 1 | 8 | 0 | 0 | 1 | 10 | 2.80% |
| NOTCH2 | 5 | 4 | 0 | 1 | 0 | 10 | 2.80% |
| OBSCN | 0 | 6 | 1 | 2 | 1 | 10 | 2.80% |
| PIK3CA | 5 | 5 | 0 | 0 | 0 | 10 | 2.80% |
| PREX2 | 8 | 2 | 0 | 0 | 0 | 10 | 2.80% |
| PRKACA | 9 | 0 | 0 | 0 | 1 | 10 | 2.80% |
| PRKDC | 10 | 0 | 0 | 0 | 0 | 10 | 2.80% |
| PTK2 | 10 | 0 | 0 | 0 | 0 | 10 | 2.80% |
| SETD2 | 0 | 6 | 0 | 4 | 0 | 10 | 2.80% |
| TNFSF13B | 10 | 0 | 0 | 0 | 0 | 10 | 2.80% |
| JUN | 9 | 0 | 0 | 0 | 0 | 9 | 2.52% |
| AKT1 | 8 | 1 | 0 | 0 | 0 | 9 | 2.52% |
| BCL2L2 | 9 | 0 | 0 | 0 | 0 | 9 | 2.52% |
| BIRC5 | 8 | 1 | 0 | 0 | 0 | 9 | 2.52% |
| CHD4 | 7 | 0 | 0 | 1 | 1 | 9 | 2.52% |
| CREBBP | 1 | 4 | 0 | 2 | 2 | 9 | 2.52% |
| ETV1 | 7 | 2 | 0 | 0 | 0 | 9 | 2.52% |
| GRIN2A | 0 | 8 | 0 | 0 | 1 | 9 | 2.52% |
| NOTCH3 | 5 | 1 | 0 | 2 | 1 | 9 | 2.52% |
| NTRK3 | 1 | 2 | 0 | 0 | 6 | 9 | 2.52% |
| SPTA1 | 0 | 8 | 0 | 1 | 0 | 9 | 2.52% |
| BRD4 | 6 | 1 | 0 | 0 | 1 | 8 | 2.24% |
| CIC | 0 | 6 | 0 | 0 | 2 | 8 | 2.24% |
| FAT1 | 1 | 4 | 3 | 0 | 0 | 8 | 2.24% |
| LRP2 | 2 | 6 | 0 | 0 | 0 | 8 | 2.24% |
| PRKAR1A | 8 | 0 | 0 | 0 | 0 | 8 | 2.24% |
| RET | 4 | 2 | 0 | 0 | 2 | 8 | 2.24% |
| TYK2 | 4 | 4 | 0 | 0 | 0 | 8 | 2.24% |
| WNK1 | 8 | 0 | 0 | 0 | 0 | 8 | 2.24% |
| ATM | 0 | 6 | 0 | 1 | 0 | 7 | 1.96% |
| CD274 | 7 | 0 | 0 | 0 | 0 | 7 | 1.96% |
| CFTR | 2 | 3 | 0 | 2 | 0 | 7 | 1.96% |
| EGFR | 2 | 5 | 0 | 0 | 0 | 7 | 1.96% |
| ERG | 5 | 1 | 0 | 0 | 1 | 7 | 1.96% |
| ETV6 | 0 | 1 | 0 | 1 | 5 | 7 | 1.96% |
| FAT4 | 0 | 6 | 0 | 1 | 0 | 7 | 1.96% |
| FGF10 | 7 | 0 | 0 | 0 | 0 | 7 | 1.96% |
| FLCN | 0 | 5 | 0 | 0 | 2 | 7 | 1.96% |
| HGF | 3 | 4 | 0 | 0 | 0 | 7 | 1.96% |
| JAK3 | 3 | 2 | 0 | 0 | 2 | 7 | 1.96% |
| KAT6A | 7 | 0 | 0 | 0 | 0 | 7 | 1.96% |
| MAGI2 | 0 | 3 | 3 | 0 | 1 | 7 | 1.96% |
| PRKCI | 6 | 1 | 0 | 0 | 0 | 7 | 1.96% |
| STK24 | 6 | 1 | 0 | 0 | 0 | 7 | 1.96% |
| TBX3 | 4 | 3 | 0 | 0 | 0 | 7 | 1.96% |
| YES1 | 6 | 1 | 0 | 0 | 0 | 7 | 1.96% |
| ABL1 | 0 | 5 | 0 | 0 | 1 | 6 | 1.68% |
| ALK | 0 | 4 | 0 | 0 | 2 | 6 | 1.68% |
| CUL4A | 6 | 0 | 0 | 0 | 0 | 6 | 1.68% |
| DEF6 | 6 | 0 | 0 | 0 | 0 | 6 | 1.68% |
| EED | 2 | 0 | 4 | 0 | 0 | 6 | 1.68% |
| EPHB1 | 0 | 5 | 0 | 1 | 0 | 6 | 1.68% |
| HSP90AA1 | 6 | 0 | 0 | 0 | 0 | 6 | 1.68% |
| KDM5C | 4 | 1 | 0 | 1 | 0 | 6 | 1.68% |
| LYN | 6 | 0 | 0 | 0 | 0 | 6 | 1.68% |
| MAPK1 | 6 | 0 | 0 | 0 | 0 | 6 | 1.68% |
| NF2 | 0 | 2 | 1 | 2 | 1 | 6 | 1.68% |
| NFE2L2 | 5 | 1 | 0 | 0 | 0 | 6 | 1.68% |
| NFIB | 6 | 0 | 0 | 0 | 0 | 6 | 1.68% |
| NRG1 | 3 | 2 | 0 | 0 | 1 | 6 | 1.68% |
| PKN1 | 6 | 0 | 0 | 0 | 0 | 6 | 1.68% |
| POLB | 6 | 0 | 0 | 0 | 0 | 6 | 1.68% |
| TRIO | 3 | 3 | 0 | 0 | 0 | 6 | 1.68% |
| ZNF217 | 5 | 1 | 0 | 0 | 0 | 6 | 1.68% |
| ANTXR2 | 5 | 0 | 0 | 0 | 0 | 5 | 1.40% |
| APC | 0 | 3 | 0 | 2 | 0 | 5 | 1.40% |
| ARAF | 5 | 0 | 0 | 0 | 0 | 5 | 1.40% |
| ARID1A | 0 | 5 | 0 | 0 | 0 | 5 | 1.40% |
| ASXL1 | 0 | 3 | 0 | 1 | 1 | 5 | 1.40% |
| AURKA | 5 | 0 | 0 | 0 | 0 | 5 | 1.40% |
| BCR | 3 | 0 | 0 | 0 | 2 | 5 | 1.40% |
| BLK | 3 | 2 | 0 | 0 | 0 | 5 | 1.40% |
| BRAF | 2 | 0 | 0 | 0 | 3 | 5 | 1.40% |
| CRKL | 5 | 0 | 0 | 0 | 0 | 5 | 1.40% |
| DDR1 | 5 | 0 | 0 | 0 | 0 | 5 | 1.40% |
| DDR2 | 5 | 0 | 0 | 0 | 0 | 5 | 1.40% |
| ERCC5 | 5 | 0 | 0 | 0 | 0 | 5 | 1.40% |
| FANCA | 0 | 5 | 0 | 0 | 0 | 5 | 1.40% |
| GATA4 | 3 | 1 | 1 | 0 | 0 | 5 | 1.40% |
| GLI2 | 3 | 2 | 0 | 0 | 0 | 5 | 1.40% |
| GLI3 | 3 | 2 | 0 | 0 | 0 | 5 | 1.40% |
| IDH2 | 2 | 3 | 0 | 0 | 0 | 5 | 1.40% |
| LZTR1 | 4 | 1 | 0 | 0 | 0 | 5 | 1.40% |
| MAP3K1 | 2 | 1 | 0 | 2 | 0 | 5 | 1.40% |
| MECOM | 4 | 1 | 0 | 0 | 0 | 5 | 1.40% |
| NPM1 | 5 | 0 | 0 | 0 | 0 | 5 | 1.40% |
| PAX5 | 3 | 2 | 0 | 0 | 0 | 5 | 1.40% |
| PDCD1LG2 | 5 | 0 | 0 | 0 | 0 | 5 | 1.40% |
| PHOX2B | 5 | 0 | 0 | 0 | 0 | 5 | 1.40% |
| POLE | 0 | 5 | 0 | 0 | 0 | 5 | 1.40% |
| PTCH1 | 0 | 2 | 0 | 1 | 2 | 5 | 1.40% |
| RIT1 | 5 | 0 | 0 | 0 | 0 | 5 | 1.40% |
| SLIT2 | 0 | 3 | 0 | 1 | 1 | 5 | 1.40% |
| SMAD2 | 3 | 1 | 0 | 1 | 0 | 5 | 1.40% |
| SMAD3 | 3 | 1 | 0 | 1 | 0 | 5 | 1.40% |
| SOX9 | 4 | 0 | 0 | 1 | 0 | 5 | 1.40% |
| TMPRSS2 | 4 | 1 | 0 | 0 | 0 | 5 | 1.40% |
| TSC2 | 0 | 4 | 0 | 0 | 1 | 5 | 1.40% |
| ZNF703 | 4 | 1 | 0 | 0 | 0 | 5 | 1.40% |
| ADGRA2 | 4 | 0 | 0 | 0 | 0 | 4 | 1.12% |
| ATR | 0 | 4 | 0 | 0 | 0 | 4 | 1.12% |
| BRCA2 | 0 | 1 | 1 | 1 | 1 | 4 | 1.12% |
| BUB1 | 1 | 3 | 0 | 0 | 0 | 4 | 1.12% |
| CHEK2 | 2 | 2 | 0 | 0 | 0 | 4 | 1.12% |
| DDIT3 | 4 | 0 | 0 | 0 | 0 | 4 | 1.12% |
| DOT1L | 0 | 4 | 0 | 0 | 0 | 4 | 1.12% |
| EP300 | 0 | 2 | 0 | 1 | 1 | 4 | 1.12% |
| EWSR1 | 0 | 2 | 0 | 0 | 2 | 4 | 1.12% |
| EZH2 | 4 | 0 | 0 | 0 | 0 | 4 | 1.12% |
| FGF4 | 3 | 0 | 0 | 0 | 1 | 4 | 1.12% |
| FGF5 | 4 | 0 | 0 | 0 | 0 | 4 | 1.12% |
| FGFR2 | 1 | 1 | 0 | 0 | 2 | 4 | 1.12% |
| FGFR3 | 0 | 2 | 0 | 0 | 2 | 4 | 1.12% |
| FLI1 | 4 | 0 | 0 | 0 | 0 | 4 | 1.12% |
| FLT4 | 2 | 2 | 0 | 0 | 0 | 4 | 1.12% |
| FNDC3B | 3 | 1 | 0 | 0 | 0 | 4 | 1.12% |
| FYN | 2 | 1 | 0 | 0 | 1 | 4 | 1.12% |
| GNA11 | 3 | 0 | 0 | 0 | 1 | 4 | 1.12% |
| GRM3 | 1 | 3 | 0 | 0 | 0 | 4 | 1.12% |
| IKBKE | 1 | 3 | 0 | 0 | 0 | 4 | 1.12% |
| INHBA | 2 | 2 | 0 | 0 | 0 | 4 | 1.12% |
| KEAP1 | 0 | 3 | 0 | 0 | 1 | 4 | 1.12% |
| MAP2K1 | 3 | 1 | 0 | 0 | 0 | 4 | 1.12% |
| MCF2L | 3 | 1 | 0 | 0 | 0 | 4 | 1.12% |
| MED12 | 0 | 2 | 0 | 2 | 0 | 4 | 1.12% |
| MET | 4 | 0 | 0 | 0 | 0 | 4 | 1.12% |
| MST1R | 0 | 4 | 0 | 0 | 0 | 4 | 1.12% |
| MYB | 3 | 1 | 0 | 0 | 0 | 4 | 1.12% |
| NCOA2 | 0 | 1 | 0 | 0 | 3 | 4 | 1.12% |
| NOTCH1 | 0 | 3 | 0 | 0 | 1 | 4 | 1.12% |
| NSD1 | 2 | 1 | 0 | 0 | 1 | 4 | 1.12% |
| PDGFRB | 1 | 3 | 0 | 0 | 0 | 4 | 1.12% |
| PIK3CG | 1 | 3 | 0 | 0 | 0 | 4 | 1.12% |
| PIK3R2 | 4 | 0 | 0 | 0 | 0 | 4 | 1.12% |
| RHBDF2 | 4 | 0 | 0 | 0 | 0 | 4 | 1.12% |
| RHEB | 4 | 0 | 0 | 0 | 0 | 4 | 1.12% |
| ROCK2 | 3 | 0 | 0 | 0 | 1 | 4 | 1.12% |
| SMARCA2 | 2 | 1 | 1 | 0 | 0 | 4 | 1.12% |
| STAT6 | 4 | 0 | 0 | 0 | 0 | 4 | 1.12% |
| STK11 | 0 | 1 | 0 | 0 | 3 | 4 | 1.12% |
| SYK | 0 | 3 | 0 | 0 | 1 | 4 | 1.12% |
| TFE3 | 4 | 0 | 0 | 0 | 0 | 4 | 1.12% |
| TIE1 | 1 | 3 | 0 | 0 | 0 | 4 | 1.12% |
| TOP1 | 4 | 0 | 0 | 0 | 0 | 4 | 1.12% |
| TSC1 | 0 | 3 | 0 | 0 | 1 | 4 | 1.12% |
| WT1 | 0 | 2 | 0 | 2 | 0 | 4 | 1.12% |
| AFF3 | 3 | 0 | 0 | 0 | 0 | 3 | 0.84% |
| APEX1 | 2 | 1 | 0 | 0 | 0 | 3 | 0.84% |
| BCL2 | 2 | 1 | 0 | 0 | 0 | 3 | 0.84% |
| BMX | 3 | 0 | 0 | 0 | 0 | 3 | 0.84% |
| BRCA1 | 0 | 0 | 0 | 0 | 3 | 3 | 0.84% |
| CASP8 | 0 | 0 | 1 | 2 | 0 | 3 | 0.84% |
| CCND1 | 3 | 0 | 0 | 0 | 0 | 3 | 0.84% |
| CD1C | 1 | 1 | 0 | 1 | 0 | 3 | 0.84% |
| CD1E | 1 | 2 | 0 | 0 | 0 | 3 | 0.84% |
| CD36 | 0 | 3 | 0 | 0 | 0 | 3 | 0.84% |
| CDK6 | 3 | 0 | 0 | 0 | 0 | 3 | 0.84% |
| CEBPA | 3 | 0 | 0 | 0 | 0 | 3 | 0.84% |
| CTLA4 | 0 | 1 | 2 | 0 | 0 | 3 | 0.84% |
| CTNNA2 | 0 | 3 | 0 | 0 | 0 | 3 | 0.84% |
| DNMT3B | 2 | 1 | 0 | 0 | 0 | 3 | 0.84% |
| DPYD | 0 | 3 | 0 | 0 | 0 | 3 | 0.84% |
| ECT2 | 3 | 0 | 0 | 0 | 0 | 3 | 0.84% |
| EPHA3 | 2 | 1 | 0 | 0 | 0 | 3 | 0.84% |
| EPHA5 | 2 | 1 | 0 | 0 | 0 | 3 | 0.84% |
| EPHA8 | 0 | 3 | 0 | 0 | 0 | 3 | 0.84% |
| ESR1 | 0 | 3 | 0 | 0 | 0 | 3 | 0.84% |
| FAT3 | 0 | 2 | 0 | 1 | 0 | 3 | 0.84% |
| FGF19 | 3 | 0 | 0 | 0 | 0 | 3 | 0.84% |
| FLT3 | 1 | 2 | 0 | 0 | 0 | 3 | 0.84% |
| FUS | 1 | 0 | 0 | 1 | 1 | 3 | 0.84% |
| GATA1 | 2 | 1 | 0 | 0 | 0 | 3 | 0.84% |
| IDO1 | 3 | 0 | 0 | 0 | 0 | 3 | 0.84% |
| IRS2 | 3 | 0 | 0 | 0 | 0 | 3 | 0.84% |
| KEL | 0 | 2 | 1 | 0 | 0 | 3 | 0.84% |
| MAP2K2 | 3 | 0 | 0 | 0 | 0 | 3 | 0.84% |
| MSH2 | 0 | 2 | 0 | 1 | 0 | 3 | 0.84% |
| MYH11 | 0 | 3 | 0 | 0 | 0 | 3 | 0.84% |
| NOTCH4 | 2 | 1 | 0 | 0 | 0 | 3 | 0.84% |
| PBRM1 | 0 | 1 | 0 | 1 | 1 | 3 | 0.84% |
| PBX1 | 1 | 0 | 0 | 0 | 2 | 3 | 0.84% |
| PDGFB | 1 | 1 | 0 | 0 | 1 | 3 | 0.84% |
| PDK1 | 3 | 0 | 0 | 0 | 0 | 3 | 0.84% |
| PIK3C2G | 0 | 1 | 1 | 1 | 0 | 3 | 0.84% |
| PTPRO | 0 | 2 | 0 | 0 | 1 | 3 | 0.84% |
| PTPRT | 0 | 3 | 0 | 0 | 0 | 3 | 0.84% |
| RARA | 0 | 2 | 0 | 0 | 1 | 3 | 0.84% |
| RPTOR | 3 | 0 | 0 | 0 | 0 | 3 | 0.84% |
| RXRA | 0 | 1 | 0 | 0 | 2 | 3 | 0.84% |
| SGK1 | 3 | 0 | 0 | 0 | 0 | 3 | 0.84% |
| SMARCA4 | 1 | 1 | 0 | 1 | 0 | 3 | 0.84% |
| SPEN | 0 | 2 | 0 | 0 | 1 | 3 | 0.84% |
| SSX1 | 2 | 1 | 0 | 0 | 0 | 3 | 0.84% |
| STAG2 | 0 | 2 | 0 | 1 | 0 | 3 | 0.84% |
| SUZ12 | 0 | 0 | 0 | 3 | 0 | 3 | 0.84% |
| TCF7L2 | 1 | 1 | 0 | 1 | 0 | 3 | 0.84% |
| TERC | 3 | 0 | 0 | 0 | 0 | 3 | 0.84% |
| TET1 | 0 | 2 | 0 | 1 | 0 | 3 | 0.84% |
| TNFSF11 | 0 | 3 | 0 | 0 | 0 | 3 | 0.84% |
| USP6 | 0 | 2 | 0 | 0 | 1 | 3 | 0.84% |
| VGLL3 | 2 | 1 | 0 | 0 | 0 | 3 | 0.84% |
| ABL2 | 2 | 0 | 0 | 0 | 0 | 2 | 0.56% |
| ACVR1B | 2 | 0 | 0 | 0 | 0 | 2 | 0.56% |
| ACVR2A | 2 | 0 | 0 | 0 | 0 | 2 | 0.56% |
| ADAM29 | 0 | 2 | 0 | 0 | 0 | 2 | 0.56% |
| AKT2 | 2 | 0 | 0 | 0 | 0 | 2 | 0.56% |
| ATF1 | 2 | 0 | 0 | 0 | 0 | 2 | 0.56% |
| AXIN1 | 0 | 2 | 0 | 0 | 0 | 2 | 0.56% |
| BAP1 | 0 | 1 | 0 | 1 | 0 | 2 | 0.56% |
| BCL6 | 1 | 1 | 0 | 0 | 0 | 2 | 0.56% |
| BIRC3 | 1 | 1 | 0 | 0 | 0 | 2 | 0.56% |
| CD1A | 1 | 1 | 0 | 0 | 0 | 2 | 0.56% |
| CD22 | 2 | 0 | 0 | 0 | 0 | 2 | 0.56% |
| CDH1 | 0 | 2 | 0 | 0 | 0 | 2 | 0.56% |
| CDKN1A | 0 | 2 | 0 | 0 | 0 | 2 | 0.56% |
| CDKN1B | 0 | 0 | 0 | 0 | 2 | 2 | 0.56% |
| CHD2 | 0 | 2 | 0 | 0 | 0 | 2 | 0.56% |
| CHEK1 | 0 | 1 | 0 | 1 | 0 | 2 | 0.56% |
| CRLF2 | 2 | 0 | 0 | 0 | 0 | 2 | 0.56% |
| CTNNA3 | 0 | 1 | 0 | 1 | 0 | 2 | 0.56% |
| CTNNB1 | 0 | 2 | 0 | 0 | 0 | 2 | 0.56% |
| DNMT3A | 1 | 1 | 0 | 0 | 0 | 2 | 0.56% |
| E2F3 | 1 | 1 | 0 | 0 | 0 | 2 | 0.56% |
| EPHA2 | 0 | 1 | 0 | 0 | 1 | 2 | 0.56% |
| EPHA7 | 0 | 2 | 0 | 0 | 0 | 2 | 0.56% |
| ERBB3 | 1 | 1 | 0 | 0 | 0 | 2 | 0.56% |
| ERBB4 | 0 | 2 | 0 | 0 | 0 | 2 | 0.56% |
| EZR | 2 | 0 | 0 | 0 | 0 | 2 | 0.56% |
| FAS | 0 | 0 | 2 | 0 | 0 | 2 | 0.56% |
| FBXO31 | 1 | 0 | 0 | 0 | 1 | 2 | 0.56% |
| FGF14 | 2 | 0 | 0 | 0 | 0 | 2 | 0.56% |
| FGF3 | 2 | 0 | 0 | 0 | 0 | 2 | 0.56% |
| FGFR4 | 1 | 1 | 0 | 0 | 0 | 2 | 0.56% |
| FGR | 1 | 1 | 0 | 0 | 0 | 2 | 0.56% |
| FOXO1 | 0 | 1 | 1 | 0 | 0 | 2 | 0.56% |
| GATA3 | 1 | 1 | 0 | 0 | 0 | 2 | 0.56% |
| GNAQ | 2 | 0 | 0 | 0 | 0 | 2 | 0.56% |
| HCK | 1 | 1 | 0 | 0 | 0 | 2 | 0.56% |
| HNF1A | 0 | 2 | 0 | 0 | 0 | 2 | 0.56% |
| HRAS | 0 | 1 | 1 | 0 | 0 | 2 | 0.56% |
| IGF2 | 0 | 2 | 0 | 0 | 0 | 2 | 0.56% |
| IRF2 | 0 | 0 | 2 | 0 | 0 | 2 | 0.56% |
| IRF4 | 2 | 0 | 0 | 0 | 0 | 2 | 0.56% |
| ITK | 2 | 0 | 0 | 0 | 0 | 2 | 0.56% |
| KMT2A | 1 | 1 | 0 | 0 | 0 | 2 | 0.56% |
| LOXL2 | 0 | 2 | 0 | 0 | 0 | 2 | 0.56% |
| MACC1 | 2 | 0 | 0 | 0 | 0 | 2 | 0.56% |
| MEN1 | 0 | 0 | 0 | 2 | 0 | 2 | 0.56% |
| MERTK | 2 | 0 | 0 | 0 | 0 | 2 | 0.56% |
| MITF | 1 | 1 | 0 | 0 | 0 | 2 | 0.56% |
| MLH1 | 0 | 1 | 0 | 1 | 0 | 2 | 0.56% |
| MS4A1 | 2 | 0 | 0 | 0 | 0 | 2 | 0.56% |
| MTOR | 0 | 2 | 0 | 0 | 0 | 2 | 0.56% |
| MUTYH | 0 | 0 | 0 | 2 | 0 | 2 | 0.56% |
| MYBL1 | 0 | 1 | 0 | 0 | 1 | 2 | 0.56% |
| MYCN | 2 | 0 | 0 | 0 | 0 | 2 | 0.56% |
| NRAS | 1 | 1 | 0 | 0 | 0 | 2 | 0.56% |
| NSD3 | 2 | 0 | 0 | 0 | 0 | 2 | 0.56% |
| NTRK1 | 2 | 0 | 0 | 0 | 0 | 2 | 0.56% |
| NTRK2 | 1 | 1 | 0 | 0 | 0 | 2 | 0.56% |
| PAX3 | 0 | 2 | 0 | 0 | 0 | 2 | 0.56% |
| PHF6 | 0 | 2 | 0 | 0 | 0 | 2 | 0.56% |
| PIK3CD | 0 | 2 | 0 | 0 | 0 | 2 | 0.56% |
| PIK3R1 | 0 | 1 | 0 | 0 | 1 | 2 | 0.56% |
| PMS2 | 0 | 2 | 0 | 0 | 0 | 2 | 0.56% |
| PTK6 | 2 | 0 | 0 | 0 | 0 | 2 | 0.56% |
| PTPN11 | 0 | 2 | 0 | 0 | 0 | 2 | 0.56% |
| RAD51C | 0 | 1 | 0 | 0 | 1 | 2 | 0.56% |
| RAD54L | 0 | 2 | 0 | 0 | 0 | 2 | 0.56% |
| ROS1 | 0 | 2 | 0 | 0 | 0 | 2 | 0.56% |
| SDC4 | 2 | 0 | 0 | 0 | 0 | 2 | 0.56% |
| SLC6A2 | 0 | 2 | 0 | 0 | 0 | 2 | 0.56% |
| SLX4 | 0 | 1 | 0 | 0 | 1 | 2 | 0.56% |
| SMARCB1 | 0 | 2 | 0 | 0 | 0 | 2 | 0.56% |
| SND1 | 1 | 1 | 0 | 0 | 0 | 2 | 0.56% |
| SOX2 | 2 | 0 | 0 | 0 | 0 | 2 | 0.56% |
| SRGAP1 | 2 | 0 | 0 | 0 | 0 | 2 | 0.56% |
| SS18 | 0 | 1 | 0 | 0 | 1 | 2 | 0.56% |
| SUFU | 0 | 1 | 0 | 0 | 1 | 2 | 0.56% |
| TAF1 | 1 | 1 | 0 | 0 | 0 | 2 | 0.56% |
| TCF3 | 1 | 1 | 0 | 0 | 0 | 2 | 0.56% |
| TEK | 2 | 0 | 0 | 0 | 0 | 2 | 0.56% |
| TP63 | 2 | 0 | 0 | 0 | 0 | 2 | 0.56% |
| TRAF7 | 0 | 2 | 0 | 0 | 0 | 2 | 0.56% |
| TSHR | 0 | 2 | 0 | 0 | 0 | 2 | 0.56% |
| VAV1 | 0 | 2 | 0 | 0 | 0 | 2 | 0.56% |
| WEE1 | 1 | 0 | 0 | 1 | 0 | 2 | 0.56% |
| WEE2 | 2 | 0 | 0 | 0 | 0 | 2 | 0.56% |
| ABCB1 | 0 | 0 | 0 | 1 | 0 | 1 | 0.28% |
| AKT3 | 0 | 1 | 0 | 0 | 0 | 1 | 0.28% |
| AMER1 | 0 | 1 | 0 | 0 | 0 | 1 | 0.28% |
| APOBEC3B | 1 | 0 | 0 | 0 | 0 | 1 | 0.28% |
| AR | 0 | 1 | 0 | 0 | 0 | 1 | 0.28% |
| ARAP3 | 0 | 1 | 0 | 0 | 0 | 1 | 0.28% |
| ARHGEF10 | 0 | 0 | 1 | 0 | 0 | 1 | 0.28% |
| ARHGEF17 | 1 | 0 | 0 | 0 | 0 | 1 | 0.28% |
| ARHGEF3 | 0 | 1 | 0 | 0 | 0 | 1 | 0.28% |
| ARID1B | 0 | 1 | 0 | 0 | 0 | 1 | 0.28% |
| ARID2 | 0 | 0 | 0 | 0 | 1 | 1 | 0.28% |
| AXIN2 | 0 | 0 | 0 | 0 | 1 | 1 | 0.28% |
| AXL | 1 | 0 | 0 | 0 | 0 | 1 | 0.28% |
| BARD1 | 1 | 0 | 0 | 0 | 0 | 1 | 0.28% |
| BCL2L1 | 1 | 0 | 0 | 0 | 0 | 1 | 0.28% |
| BCL7A | 0 | 0 | 0 | 0 | 1 | 1 | 0.28% |
| BCORL1 | 0 | 1 | 0 | 0 | 0 | 1 | 0.28% |
| BLM | 0 | 1 | 0 | 0 | 0 | 1 | 0.28% |
| BMPR1A | 1 | 0 | 0 | 0 | 0 | 1 | 0.28% |
| BRIP1 | 0 | 0 | 0 | 0 | 1 | 1 | 0.28% |
| BTG1 | 0 | 0 | 0 | 1 | 0 | 1 | 0.28% |
| CBFB | 0 | 0 | 0 | 0 | 1 | 1 | 0.28% |
| CBL | 0 | 0 | 1 | 0 | 0 | 1 | 0.28% |
| CD1B | 1 | 0 | 0 | 0 | 0 | 1 | 0.28% |
| CD1D | 1 | 0 | 0 | 0 | 0 | 1 | 0.28% |
| CD70 | 1 | 0 | 0 | 0 | 0 | 1 | 0.28% |
| CD74 | 1 | 0 | 0 | 0 | 0 | 1 | 0.28% |
| CDK12 | 1 | 0 | 0 | 0 | 0 | 1 | 0.28% |
| CDK2 | 1 | 0 | 0 | 0 | 0 | 1 | 0.28% |
| CDK8 | 1 | 0 | 0 | 0 | 0 | 1 | 0.28% |
| CDKN2C | 0 | 0 | 0 | 1 | 0 | 1 | 0.28% |
| COL2A1 | 0 | 0 | 0 | 1 | 0 | 1 | 0.28% |
| CSF1 | 0 | 1 | 0 | 0 | 0 | 1 | 0.28% |
| CSF3R | 0 | 1 | 0 | 0 | 0 | 1 | 0.28% |
| CSK | 0 | 1 | 0 | 0 | 0 | 1 | 0.28% |
| CTCF | 1 | 0 | 0 | 0 | 0 | 1 | 0.28% |
| CUL3 | 0 | 1 | 0 | 0 | 0 | 1 | 0.28% |
| CXCR4 | 0 | 1 | 0 | 0 | 0 | 1 | 0.28% |
| CYLD | 0 | 0 | 0 | 1 | 0 | 1 | 0.28% |
| CYP2D6 | 0 | 1 | 0 | 0 | 0 | 1 | 0.28% |
| DAXX | 0 | 1 | 0 | 0 | 0 | 1 | 0.28% |
| DYNLL1 | 1 | 0 | 0 | 0 | 0 | 1 | 0.28% |
| EGF | 0 | 1 | 0 | 0 | 0 | 1 | 0.28% |
| EMSY | 1 | 0 | 0 | 0 | 0 | 1 | 0.28% |
| ERCC1 | 1 | 0 | 0 | 0 | 0 | 1 | 0.28% |
| ERCC2 | 0 | 1 | 0 | 0 | 0 | 1 | 0.28% |
| ETV4 | 0 | 0 | 0 | 0 | 1 | 1 | 0.28% |
| ETV5 | 1 | 0 | 0 | 0 | 0 | 1 | 0.28% |
| FANCD2 | 1 | 0 | 0 | 0 | 0 | 1 | 0.28% |
| FANCL | 0 | 0 | 0 | 0 | 1 | 1 | 0.28% |
| FANCM | 0 | 0 | 0 | 0 | 1 | 1 | 0.28% |
| FBXW7 | 0 | 1 | 0 | 0 | 0 | 1 | 0.28% |
| FEN1 | 1 | 0 | 0 | 0 | 0 | 1 | 0.28% |
| FGF18 | 1 | 0 | 0 | 0 | 0 | 1 | 0.28% |
| FGF7 | 1 | 0 | 0 | 0 | 0 | 1 | 0.28% |
| FGF9 | 0 | 1 | 0 | 0 | 0 | 1 | 0.28% |
| FLT1 | 1 | 0 | 0 | 0 | 0 | 1 | 0.28% |
| FOXM1 | 0 | 1 | 0 | 0 | 0 | 1 | 0.28% |
| GABRA6 | 1 | 0 | 0 | 0 | 0 | 1 | 0.28% |
| GALNT12 | 0 | 1 | 0 | 0 | 0 | 1 | 0.28% |
| GATA6 | 1 | 0 | 0 | 0 | 0 | 1 | 0.28% |
| HDAC2 | 1 | 0 | 0 | 0 | 0 | 1 | 0.28% |
| HMGA1 | 1 | 0 | 0 | 0 | 0 | 1 | 0.28% |
| IKZF1 | 0 | 1 | 0 | 0 | 0 | 1 | 0.28% |
| IRF1 | 0 | 0 | 0 | 1 | 0 | 1 | 0.28% |
| JAZF1 | 1 | 0 | 0 | 0 | 0 | 1 | 0.28% |
| KDM5B | 0 | 1 | 0 | 0 | 0 | 1 | 0.28% |
| KLHL6 | 1 | 0 | 0 | 0 | 0 | 1 | 0.28% |
| LMO1 | 0 | 1 | 0 | 0 | 0 | 1 | 0.28% |
| MAF | 0 | 0 | 0 | 1 | 0 | 1 | 0.28% |
| MAP3K13 | 1 | 0 | 0 | 0 | 0 | 1 | 0.28% |
| MAP4K5 | 1 | 0 | 0 | 0 | 0 | 1 | 0.28% |
| MDM4 | 1 | 0 | 0 | 0 | 0 | 1 | 0.28% |
| MEF2B | 1 | 0 | 0 | 0 | 0 | 1 | 0.28% |
| MPL | 1 | 0 | 0 | 0 | 0 | 1 | 0.28% |
| MRE11 | 1 | 0 | 0 | 0 | 0 | 1 | 0.28% |
| MSH6 | 0 | 1 | 0 | 0 | 0 | 1 | 0.28% |
| NAB2 | 0 | 0 | 0 | 0 | 1 | 1 | 0.28% |
| NBN | 0 | 1 | 0 | 0 | 0 | 1 | 0.28% |
| NECTIN4 | 1 | 0 | 0 | 0 | 0 | 1 | 0.28% |
| NET1 | 1 | 0 | 0 | 0 | 0 | 1 | 0.28% |
| NFKBIA | 0 | 0 | 0 | 0 | 1 | 1 | 0.28% |
| NKX2-1 | 1 | 0 | 0 | 0 | 0 | 1 | 0.28% |
| NR4A3 | 0 | 1 | 0 | 0 | 0 | 1 | 0.28% |
| NRG3 | 0 | 1 | 0 | 0 | 0 | 1 | 0.28% |
| NSD2 | 1 | 0 | 0 | 0 | 0 | 1 | 0.28% |
| NTHL1 | 1 | 0 | 0 | 0 | 0 | 1 | 0.28% |
| PAK3 | 1 | 0 | 0 | 0 | 0 | 1 | 0.28% |
| PALB2 | 0 | 0 | 0 | 0 | 1 | 1 | 0.28% |
| PARP4 | 0 | 0 | 0 | 0 | 1 | 1 | 0.28% |
| PIK3C2B | 1 | 0 | 0 | 0 | 0 | 1 | 0.28% |
| PIK3CB | 1 | 0 | 0 | 0 | 0 | 1 | 0.28% |
| PKD2 | 1 | 0 | 0 | 0 | 0 | 1 | 0.28% |
| POLD1 | 0 | 1 | 0 | 0 | 0 | 1 | 0.28% |
| POT1 | 1 | 0 | 0 | 0 | 0 | 1 | 0.28% |
| PPP2R2A | 1 | 0 | 0 | 0 | 0 | 1 | 0.28% |
| PRDM1 | 0 | 1 | 0 | 0 | 0 | 1 | 0.28% |
| PRKN | 0 | 1 | 0 | 0 | 0 | 1 | 0.28% |
| PRPF38B | 1 | 0 | 0 | 0 | 0 | 1 | 0.28% |
| RAD51 | 1 | 0 | 0 | 0 | 0 | 1 | 0.28% |
| RAD51B | 0 | 0 | 0 | 0 | 1 | 1 | 0.28% |
| RAD51D | 0 | 0 | 1 | 0 | 0 | 1 | 0.28% |
| RANBP2 | 0 | 0 | 0 | 1 | 0 | 1 | 0.28% |
| RASA1 | 0 | 1 | 0 | 0 | 0 | 1 | 0.28% |
| RECQL | 0 | 0 | 0 | 1 | 0 | 1 | 0.28% |
| RECQL4 | 1 | 0 | 0 | 0 | 0 | 1 | 0.28% |
| RELA | 0 | 1 | 0 | 0 | 0 | 1 | 0.28% |
| REV3L | 0 | 1 | 0 | 0 | 0 | 1 | 0.28% |
| RGS7 | 0 | 1 | 0 | 0 | 0 | 1 | 0.28% |
| RNASEL | 0 | 1 | 0 | 0 | 0 | 1 | 0.28% |
| RNF43 | 0 | 1 | 0 | 0 | 0 | 1 | 0.28% |
| ROCK1 | 1 | 0 | 0 | 0 | 0 | 1 | 0.28% |
| RUNX1 | 0 | 1 | 0 | 0 | 0 | 1 | 0.28% |
| SERPINB4 | 0 | 1 | 0 | 0 | 0 | 1 | 0.28% |
| SETBP1 | 1 | 0 | 0 | 0 | 0 | 1 | 0.28% |
| SHQ1 | 0 | 1 | 0 | 0 | 0 | 1 | 0.28% |
| SOX10 | 1 | 0 | 0 | 0 | 0 | 1 | 0.28% |
| SPINK1 | 0 | 1 | 0 | 0 | 0 | 1 | 0.28% |
| SPOP | 1 | 0 | 0 | 0 | 0 | 1 | 0.28% |
| SRC | 1 | 0 | 0 | 0 | 0 | 1 | 0.28% |
| SRMS | 0 | 1 | 0 | 0 | 0 | 1 | 0.28% |
| STAT3 | 1 | 0 | 0 | 0 | 0 | 1 | 0.28% |
| STAT4 | 1 | 0 | 0 | 0 | 0 | 1 | 0.28% |
| TACSTD2 | 1 | 0 | 0 | 0 | 0 | 1 | 0.28% |
| TGFBR1 | 0 | 1 | 0 | 0 | 0 | 1 | 0.28% |
| TGFBR2 | 0 | 1 | 0 | 0 | 0 | 1 | 0.28% |
| TNK2 | 1 | 0 | 0 | 0 | 0 | 1 | 0.28% |
| U2AF1 | 1 | 0 | 0 | 0 | 0 | 1 | 0.28% |
| UGT1A1 | 0 | 0 | 1 | 0 | 0 | 1 | 0.28% |
| VHL | 0 | 0 | 0 | 0 | 1 | 1 | 0.28% |
| WRN | 1 | 0 | 0 | 0 | 0 | 1 | 0.28% |
| XPO1 | 0 | 1 | 0 | 0 | 0 | 1 | 0.28% |
| XRCC2 | 1 | 0 | 0 | 0 | 0 | 1 | 0.28% |
| YAP1 | 1 | 0 | 0 | 0 | 0 | 1 | 0.28% |
| ZNRF3 | 0 | 0 | 0 | 1 | 0 | 1 | 0.28% |
