# Supplementary material for: A Comprehensive Understanding of the Genomic Bone Tumor Landscape: A Multicenter Prospective Study
Source: Front Oncol. 2022 Jun 8;12:835004. doi: 10.3389/fonc.2022.835004 (PMC9213736; doi:10.3389/fonc.2022.835004)
Supplement: Supplementary file 2 [file DataSheet_1.docx]

**
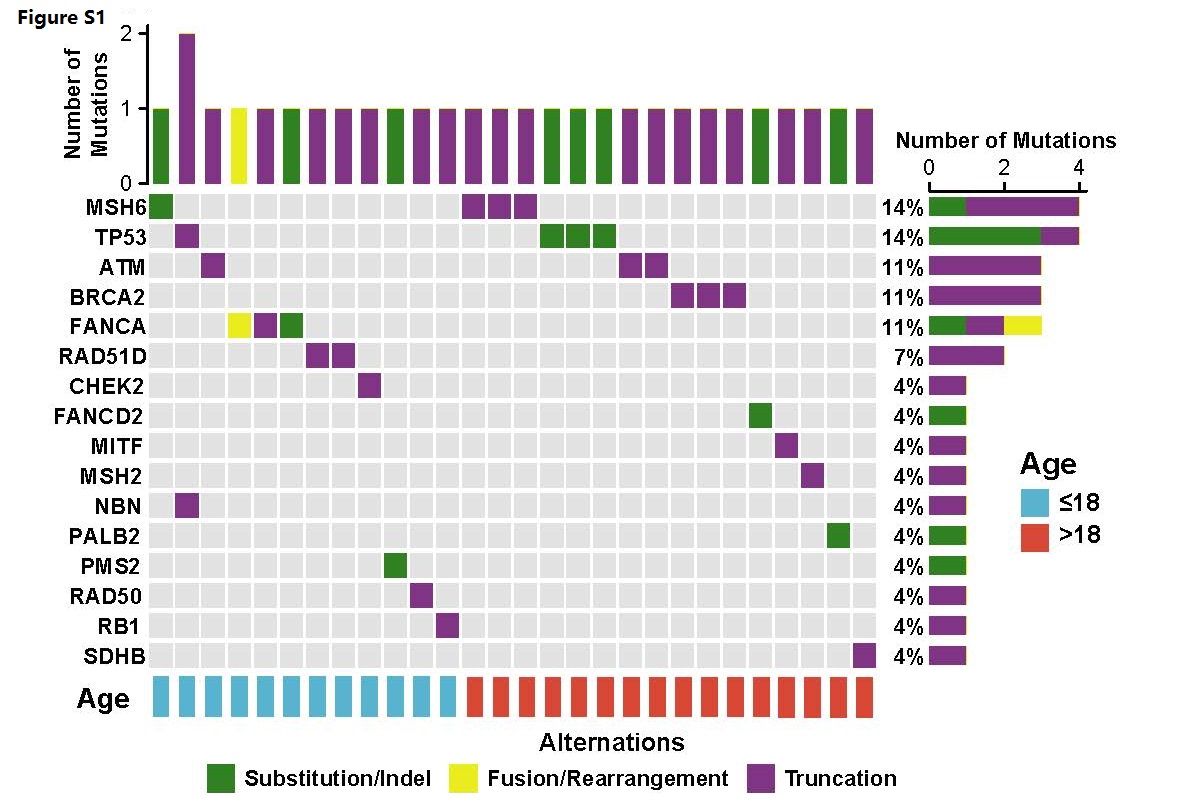
**

**Figure S1 Germline mutations of Chinese bone cancer patients.** The cohort was classified into juvenile (≤18 years, blue) and adult (>18 years, red) group based on the age of patients. The X-axis represents each case sample and the Y-axis represents each mutated gene. The bar graph on the right shows the mutation number of each gene, and the bar graph above shows the mutation number of each sample. Green represents substitution/Indel mutations, yellow represents fusion/rearrangement mutations, and purple represents truncation mutations.

**
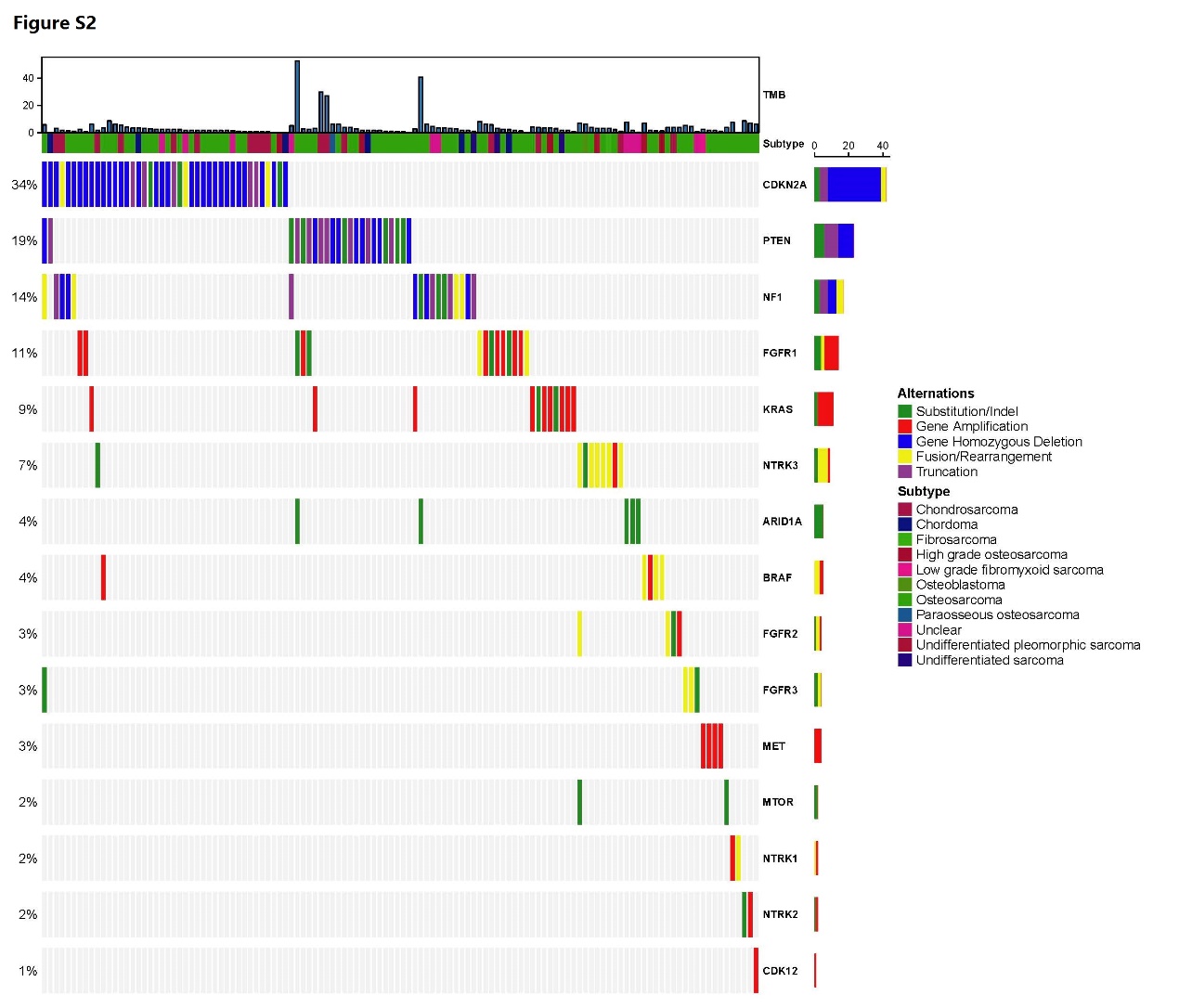
**

**Figure S2** **Mutational profiling of actionable targeted mutations in this cohort.** The X-axis represents each case sample and the Y-axis represents each mutated gene. The bar graph on the right shows the mutation number of each gene, and the bar graph above shows the mutation number of each sample. Green represents substitution/Indel mutations, red represents gene amplification mutations, blue represents gene homozygous deletion mutations, yellow represents fusion/rearrangement mutations, and purple represents truncation mutations.

**
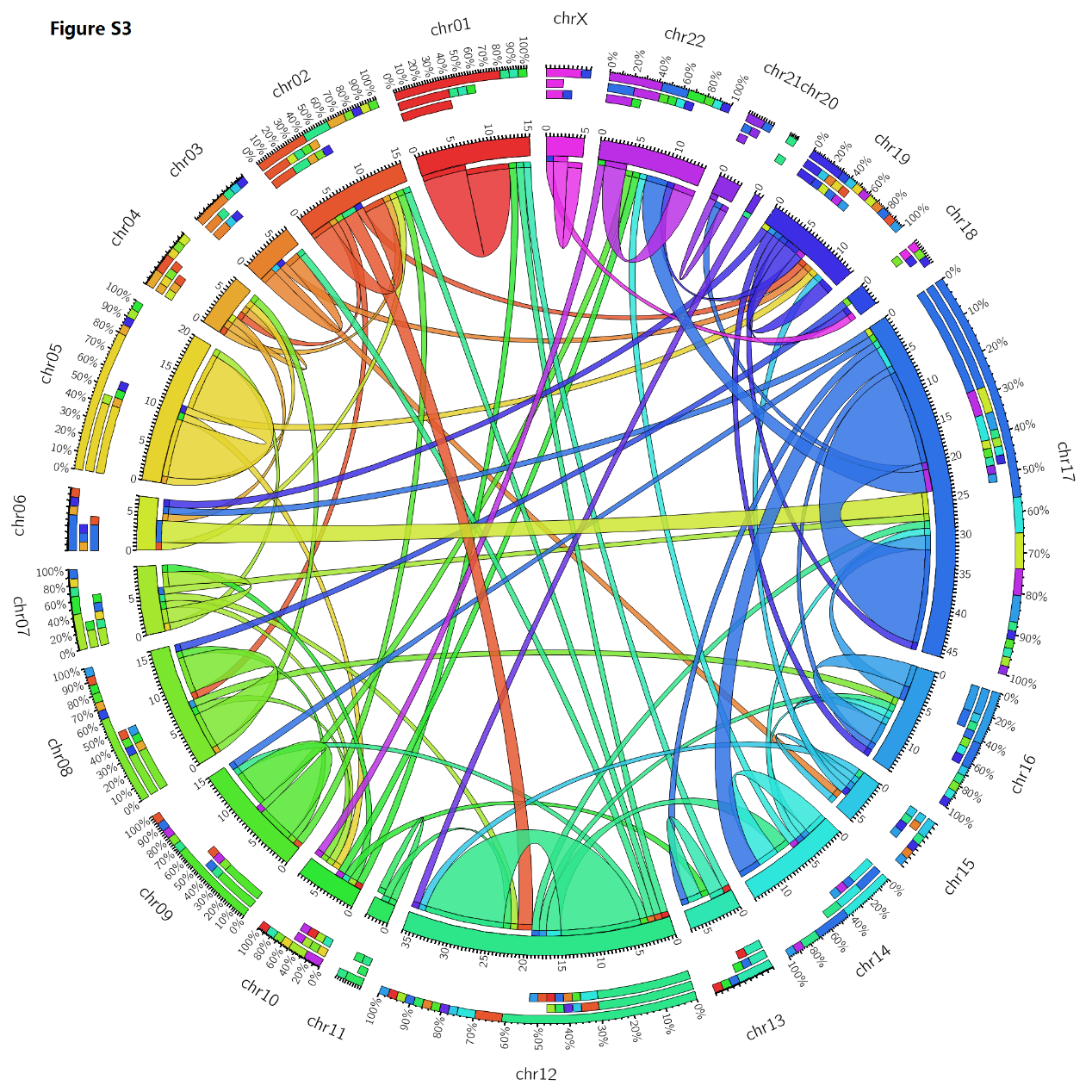
Figure S3 The profiles of gene fusion in this cohort.**  Circos Tableviewer (http://mkweb.bcgsc.ca/tableviewer/) representation as in Figure. Chromosomes are arranged circularly end-to-end. The outer circle represents the total fusion events, the proportion of upstream gene breakpoints, and the proportion of downstream gene breakpoints. The inner ring displays the fusions occurred in intrachromosomal or interchromosomal.

**
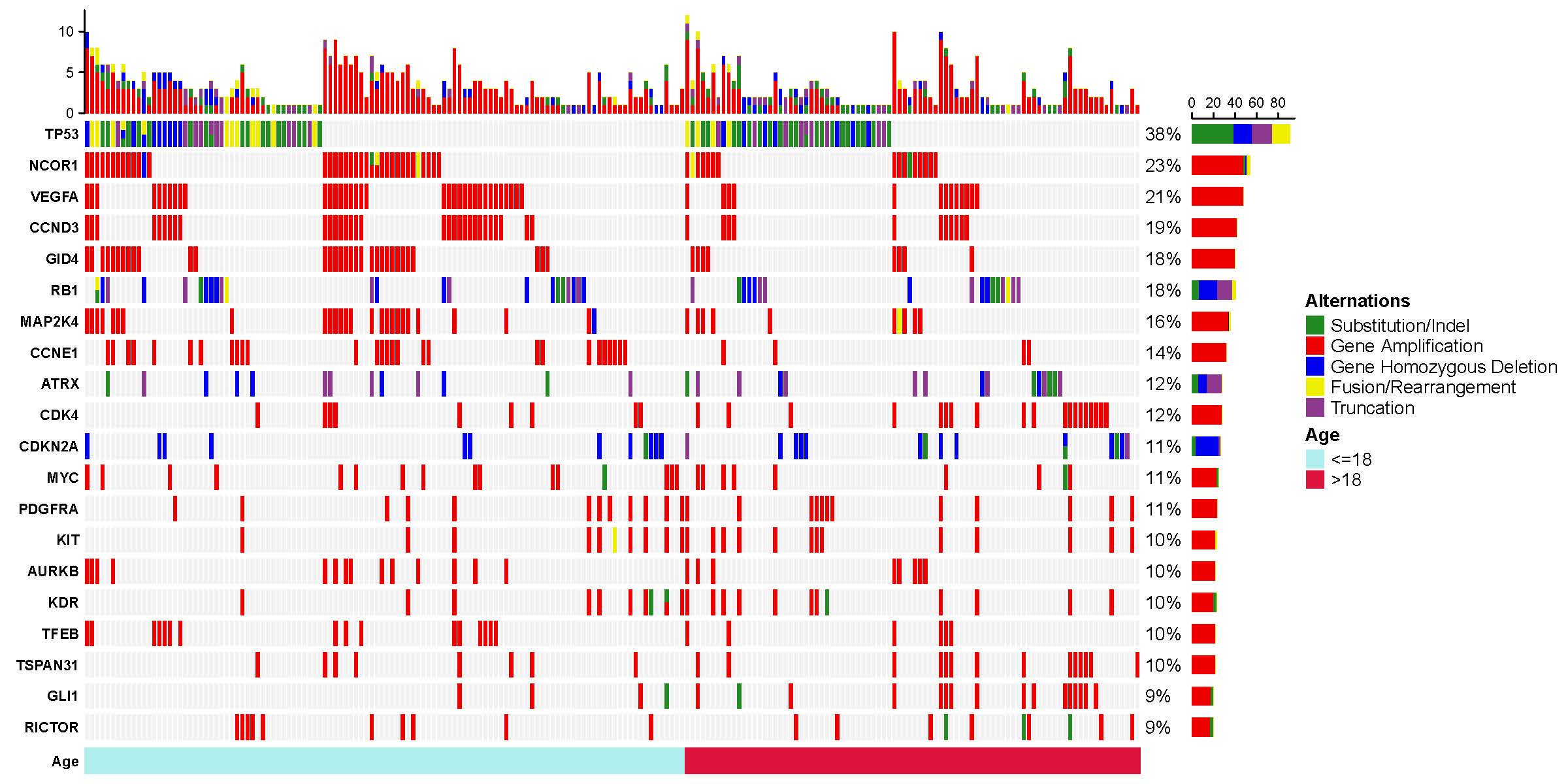
 Figure S4 Mutational profiling of osteosarcoma patients.** The cohort was classified into juvenile (≤18 years, blue) and adult (>18 years, red) group based on the age of patients. The X-axis represents each case sample and the Y-axis represents each mutated gene. The bar graph on the right shows the mutation number of each gene, and the bar graph above shows the mutation number of each sample. Green represents substitution/Indel mutations, red represents gene amplification mutations, blue represents gene homozygous deletion mutations, yellow represents fusion/rearrangement mutations, and purple represents truncation mutations.

**
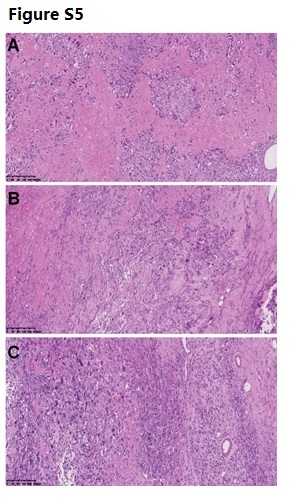
**

**Figure S5 HE staining of tumor sections.** (A) distribution of osteoid; (B) distribution of mitotic cells; (C) locally distributed bone like matrix and multinucleated giant cells.
